# Systematic review of international studies evaluating MDRD and CKD-EPI estimated glomerular filtration rate (eGFR) equations in Black adults

**DOI:** 10.1371/journal.pone.0276252

**Published:** 2022-10-18

**Authors:** Ebele M. Umeukeje, Taneya Y. Koonce, Sheila V. Kusnoor, Ifeoma I. Ulasi, Sophia Kostelanetz, Annette M. Williams, Mallory N. Blasingame, Marcia I. Epelbaum, Dario A. Giuse, Annie N. Apple, Karampreet Kaur, Tavia González Peña, Danika Barry, Leo G. Eisenstein, Cameron T. Nutt, Nunzia B. Giuse

**Affiliations:** 1 Division of Nephrology and Hypertension, Department of Medicine, Vanderbilt University Medical Center, Nashville, TN, United States of America; 2 Vanderbilt Center for Kidney Disease, Vanderbilt University Medical Center, Nashville, TN, United States of America; 3 Center for Knowledge Management, Vanderbilt University Medical Center, Nashville, TN, United States of America; 4 Department of Biomedical Informatics, Vanderbilt University Medical Center, Nashville, TN, United States of America; 5 Renal Unit, Department of Medicine, College of Medicine, University of Nigeria/University of Nigeria Teaching Hospital, Ituku-Ozalla, Nigeria; 6 Division of General Internal Medicine and Public Health, Vanderbilt University Medical Center, Nashville, TN, United States of America; 7 Vanderbilt University School of Medicine, Nashville, TN, United States of America; 8 Department of Obstetrics & Gynecology, McGaw Medical Center of Northwestern University, Chicago, IL, United States of America; 9 Department of Medicine, NYU Langone Medical Center, New York, NY, United States of America; 10 Department of Medicine, Brigham and Women’s Hospital, Boston, MA, United States of America; 11 Department of Medicine, Vanderbilt University Medical Center, Nashville, TN, United States of America; Baker IDI Heart and Diabetes Institute, AUSTRALIA

## Abstract

Use of race adjustment in estimating glomerular filtration rate (eGFR) has been challenged given concerns that it may negatively impact the clinical care of Black patients, as it results in Black patients being systematically assigned higher eGFR values than non-Black patients. We conducted a systematic review to assess how well eGFR, with and without race adjustment, estimates measured GFR (mGFR) in Black adults globally. A search across multiple databases for articles published from 1999 to May 2021 that compared eGFR to mGFR and reported outcomes by Black race was performed. We included studies that assessed eGFR using the Modification of Diet in Renal Disease (MDRD) and Chronic Kidney Disease Epidemiology Collaboration (CKD-EPI_Cr_) creatinine equations. Risk of study bias and applicability were assessed with the QUality Assessment of Diagnostic Accuracy Studies-2. Of 13,167 citations identified, 12 met the data synthesis criteria (unique patient cohorts in which eGFR was compared to mGFR with and without race adjustment). The studies included patients with and without kidney disease from Africa (n = 6), the United States (n = 3), Europe (n = 2), and Brazil (n = 1). Of 11 CKD-EPI equation studies, all assessed bias, 8 assessed accuracy, 6 assessed precision, and 5 assessed correlation/concordance. Of 7 MDRD equation studies, all assessed bias, 6 assessed accuracy, 5 assessed precision, and 3 assessed correlation/concordance. The majority of studies found that removal of race adjustment improved bias, accuracy, and precision of eGFR equations for Black adults. Risk of study bias was often unclear, but applicability concerns were low. Our systematic review supports the need for future studies to be conducted in diverse populations to assess the possibility of alternative approaches for estimating GFR. This study additionally provides systematic-level evidence for the American Society of Nephrology—National Kidney Foundation Task Force efforts to pursue other options for GFR estimation.

## Introduction

Accurate assessment of kidney function is essential for proper diagnosis, staging, and management of chronic kidney disease (CKD). The gold standard for evaluating kidney function is measured glomerular filtration rate (mGFR), which relies on infusing chemicals, such as iothalamate, into the blood and quantifying urine clearance. Estimated GFR (eGFR) is based on the measurement of serum filtration markers, such as creatinine or cystatin C, and therefore is more practical to obtain in clinical practice [1].

The Modification of Diet in Renal Disease (MDRD) and Chronic Kidney Disease Epidemiology Collaboration (CKD-EPI) equations are commonly used in the United States (U.S.) and internationally. Each includes serum creatinine measurement as a key variable for determining eGFR [2–4]. The equations were developed in large U.S. cohorts and include race, sex, and age variables [5]. Use of the race variable increases reported eGFR by 21.2% (MDRD) or 15.9% (CKD-EPI) in Black patients [3, 6].

While sex and age are biological variables, the meaning and classification of race have evolved over time. Definitions of race are often inconsistently applied, and the utility of race adjustment in non-U.S. populations is unclear [7]. The appropriateness of including race in clinical algorithms has been questioned given that race is a sociopolitical rather than biological construct and the urgent need for clinical medicine to confront structural racism in our practices [7–14].

In the U.S., the prevalence of chronic kidney disease (CKD) and end-stage renal disease is higher among Black Americans relative to White Americans [15, 16]. While genetic and social factors play a large role in kidney health disparities in the U.S., inappropriately including race in eGFR equations can increase health inequities [14, 17]. Race-adjusted glomerular filtration rate (GFR) estimates could delay clinical care dependent on eGFR thresholds, such as specialist referrals or transplantation, and impact dosing decisions [8, 9, 14].

Concerns about health equity and the lack of evidence for race adjustment led several institutions to omit race from eGFR equations [18]. Some researchers cautioned about unintended consequences, including the potential for Black patients to be overdiagnosed and overtreated, or prematurely deprescribed medications such as metformin or empagliflozin [8, 11, 14, 18]. In July 2020, the National Kidney Foundation and the American Society of Nephrology (NKF-ASN) created a task force to evaluate the inclusion of race in eGFR equations in response to growing controversy [19]. The task force ultimately recommended a new 2021 CKD-EPI creatinine equation refit without the race variable for U.S. adults [20, 21].

A systematic review of the global literature on the use of eGFR equations for Black patients is needed to inform clinical decision-making worldwide [14] and strengthen the adoption of the NKF-ASN task force recommendations. Our team conducted a systematic review to provide the highest level of evidence for assessing the use of eGFR race adjustment in Black patients. We aimed to answer the question: How well does eGFR, with and without race adjustment, predict mGFR in Black adults in the U.S. and internationally?

## Methods

### Protocol

A protocol with investigation parameters, data extraction procedures, and quality assessment processes was used to guide the review process (S1 Table). The protocol and systematic review are reported following the Preferred Reporting Items for Systematic Reviews and Meta-Analyses (PRISMA) guidelines (S2 Table) [22–24].

### Eligibility criteria

Inclusion criteria were validation and/or comparison studies that included Black adults (ages 18 and older), compared an eGFR formula to mGFR or to another eGFR formula, and were published in English, French, Spanish, or Portuguese. All research article types were eligible, including conference abstracts. Items were excluded if they did not use mGFR as a reference test as assessed by ^51^Cr-EDTA, inulin, ^125^I-iothalamate, iohexol, or ^99m^Tc-DTPA exogenous markers, did not use either an MDRD or CKD-EPI eGFR formula as an index test, did not use isotope dilution mass spectrometry (IDMS) traceable creatinine assays, or did not report outcomes by Black race. Conference abstracts published prior to 2015 were excluded as the findings would likely have been later published as journal articles. Studies reporting preliminary results were excluded when a more complete publication with the final results was identified.

### Search process

PubMed, Embase (via OVID), ScienceDirect (Elsevier), Web of Science, and Google Scholar (via the Publish or Perish software [25]) were searched for citations published after January 1, 1999. Database searches, originally conducted on October 23, 2020, were re-executed on May 6, 2021. All search results were deduplicated based on unique identifiers (e.g., digital object identifier, article title). See S1 File for the full search strategies and search process details.

### Study screening and data extraction

The abstracts and full-text of each publication were independently screened by 2 reviewers. Decisions were collected via REDCap® [26] forms created by the study team. Discordant ratings between reviewers were resolved using an independent third person. Data extraction was also conducted independently by 2 reviewers for each included study meeting eligibility criteria; a third person adjudicated non-consensus ratings. Data extracted included: citation details, country of study, study aims, total participants, population characteristics (e.g., demographics, kidney disease status), study eligibility criteria, eGFR and mGFR calculation methods, type of eGFR equation used, eGFR as assessed with and/or without race coefficient in Black adults, mGFR in Black adults, performance measure results, and statistical significance.

The main outcomes included bias (defined as the difference between eGFR and mGFR), accuracy (proportion of patients with eGFR values within a given threshold, such as 30% of mGFR, also referred to as P_30_), precision, and correlation/concordance of eGFR and mGFR. Additional outcomes were reporting of social determinants of health (e.g., income, educational level, lifestyle choices) and non-GFR determinants of creatinine: underlying co-morbidities (e.g., liver disease, anorexia, chronic illnesses), and medication use that can lead to false elevation of creatinine or interfere with creatinine measurement assays (e.g., antibiotics, chemotherapeutic agents).

### Quality assessment

Applicability and risk of study bias were assessed independently by 2 reviewers using the QUality Assessment of Diagnostic Accuracy Studies (QUADAS-2) [27]. The instrument includes “signaling” questions to assess bias in 4 domains (patient selection, index test, reference standard, and flow and timing) and questions in 3 domains (patient selection, index test, and reference standard) to assess the applicability of each study to the overall systematic review question.

In the patient selection domain, we assessed the appropriateness of the exclusion criteria relative to the aims of the specific study and against a pre-defined list of non-GFR determinants of creatinine; if exclusions were explained by these factors, we marked “yes” for the signaling question of whether the study avoided inappropriate exclusions. For pooled data studies, if it was unclear how the authors arrived at the number of participants compared with the original studies, we used a response of “unclear” for the exclusion criteria question. Concern that the included patients did not match the review question was rated as “high” if there was uncertainty that the study did not include only Black adults (e.g., participants <18 years old or of “mixed ancestry”). The index tests were defined as either the MDRD or CKD-EPI and the reference standard was mGFR using any of the following exogenous markers: ^51^Cr-EDTA, inulin, ^125^I-iothalamate, iohexol, or ^99m^Tc-DTPA. In the flow and timing domain, an appropriate interval between the index and reference tests was defined as eGFR and mGFR samples drawn within 24 hours of each other.

If all responses to the signaling questions were “yes,” we assigned a rating of “low” to the domain; if all responses were “no,” we assigned a rating of “high.” For any combination of responses (i.e., any combination of “yes,” “no,” and/or “unclear”), we assigned a rating of “unclear.” Discordant ratings between reviewers were resolved through discussion.

**Data synthesis.** Studies were grouped by the methods used to assess eGFR vs. mGFR performance. Three categories of publications were identified: studies that evaluated eGFR equations 1) with and without race adjustment, 2) with race adjustment, and 3) without race adjustment. Data synthesis was conducted only on studies that assessed eGFR equations both with and without race adjustment compared to mGFR (category 1) in order to have directly comparable eGFR performance data for the review analysis. Additionally, when multiple publications reporting outcomes data on the same patient population were identified, the publication with the most complete data set was retained for analysis. Data are presented using descriptive tables. The outcomes of bias, accuracy, precision, and agreement measures (e.g., correlations and concordance), are summarized by eGFR equation. No minimum number of studies was needed to report results.

## Results

**Fig 1:**
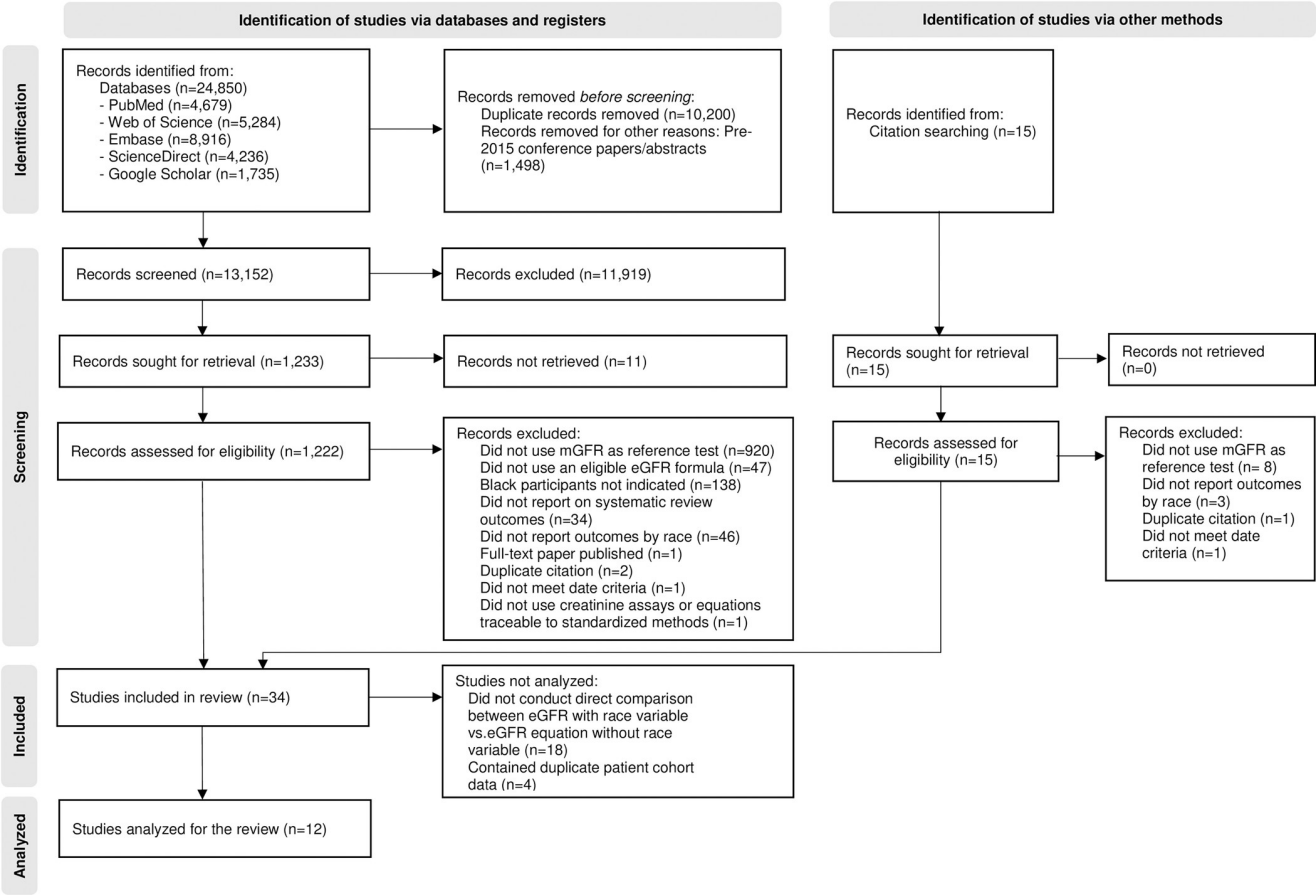
Flow diagram of study search and selection process.

In all, 24,850 results were retrieved (Fig 1) from database searches. Fifteen additional articles were identified through handsearching of 256 articles flagged for reference checking during the screening process. After removing duplicate records and pre-2015 conference abstracts, the titles and abstracts of 13,167 citations were screened. Of these, 11,919 were excluded after abstract review, and 1,190 were excluded at full-text review. Thirty-four articles met all eligibility criteria, of which, 12 met data synthesis criteria as described above.

### Study characteristics

Twelve studies evaluating eGFR equations both with and without race adjustment vs. mGFR were included in the data synthesis (Table 1). See S3 Table for the study characteristics of the remaining 22 studies identified in the review.

**Table 1:** Summary characteristics of studies included in the systematic review synthesis.

	First Author, Year	Country	Study Design/Data Source	Population	Black participants, n/N (%)	Method of race definition	eGFR equation(s)	mGFR	Outcomes reported for Black adults
1	Arlet, 2012 [35]	France	Prospective observational cohort study	Patients with sickle cell disease	64/64 (100%)^a^	Not reported	CKD-EPI_Cr_, MDRD	Iohexol	Bias, Pearson correlation
2	Atta, 2021 [34]	USA	Retrospective validation study; used patients from a previous study	Individuals who are HIV-positive and HIV-negative	327/327 (100%)^b^	Not reported	CKD-EPI_Cr_,	Iohexol	Bias, P_30_
3	Bukabau, 2019 [28]	Democratic Republic of the Congo and Ivory Coast	Cross-sectional study	Healthy individuals and individuals with CKD	494/494 (100%)	Not reported	CKD-EPI_Cr_ MDRD	Iohexol	Bias, P_30_, Precision, Correlation
4	Gama, 2021 [33]	United Kingdom	Retrospective medical record cross-sectional study	Mix: patients at a large tertiary hospital	266/1888 (14.1%)	Self-reported	CKD-EPI_Cr_, MDRD	^51^Cr-EDTA	Bias, precision, limits of agreement, P_30_
5	Holness, 2020 [38]	South Africa	Prospective validation study	Mix: patients with CKD, potential kidney donors, healthy volunteers	Mixed race: 80/80 (100%)	Self-reported	CKD-EPI_Cr_, MDRD	^99m^Tc-DTPA	Bias, IQR, RMSE, P_20_ and P_30,_ Bland-Altman limits of agreement
6	Levey, 2020 [10]	USA	Cross-sectional validation study using pooled data	Mix; included patients with and without CKD	2601/8254 (31.5%)	Self-reported or investigator-assigned	CKD-EPI_Cr_	Iothalamate	Bias, RMSE
7	Moodley, 2018 [29]	South Africa	Retrospective, observational, cross-sectional study	Inpatients and outpatients; mix of conditions	188/287 (65.5%)	Not reported	CKD-EPI_Cr_,	^99m^Tc-DTPA	Bias, P_10_, P_30_, correlation
8	Rocha, 2020 [30]	Brazil	Cross-sectional study	Patients with CKD	61/100 (61%) African Brazilians Black: n = 27 Mixed-race: n = 34	Investigator-assigned	CKD-EPI_Cr,_	^51^Cr-EDTA	Bias, precision, P_30_, concordance
9	Seape, 2016 [31]	South Africa	Cross-sectional study	Individuals with HIV who are ART-naïve	97/97 (100%)	Not reported	CKD-EPI_Cr_, MDRD	^51^Cr-EDTA	Bias, 95% limits of agreement, P_15_, P_30_
10	Van Deventer, 2008 [36]	South Africa	Prospective study	Patients with/at risk for CKD	100/100 (100%)	Not reported	MDRD	^51^Cr-EDTA	Bias, precision, RMSE, P_30_
11	Wyatt, 2013 [32]	Kenya	Cross-sectional study	Individuals who are HIV infected, ambulatory, and ART-naïve	99/99 (100%)	Self-reported	CKD-EPI_Cr_, MDRD	Iohexol (dried blood spots)^c^	Bias, P_10_, P_30,_ correlation
12	Zelnick, 2021 [37]	USA	Prospective cohort; used data from CRIC study^d^	Patients with CKD	1658/1658 (100%) Bias reported for patients with mGFR of 15 to <45 (n = 311)	Self-reported	CKD-EPI_Cr_	^125^I-iothalamate	Bias

^a^Participants were described as originating from Sub-Saharan Africa and the French West Indies, though not explicitly described as “black” by the authors.

^b^Study reports “n” as “observations,” not individual patients.

^c^Specimen type included as it is a deviation from gold standard practice

^d^Zelnick et al. report data from the CRIC study spanning 2003–2018; specifically, a subset of participants–individuals with an mGFR between 15 to 45 mL/min/1.73 m^2^. Levey et al., also included in this systematic review, includes CRIC participants as one of 10 studies pooled for the analysis. The CRIC data included in Levey et al., spans 2003–2005 only.

Abbreviations: ^99m^Tc-DTPA = technetium-99m diethylenetriamine pentaacetic acid; ^51^Cr-EDTA = chromium-51 labeled ethylenediamine tetraacetic acid; ART = antiretroviral therapy; CKD = chronic kidney disease; CKD-EPI = Chronic Kidney Disease Epidemiology Collaboration; Cr = creatinine; CRIC = Chronic Renal Insufficiency Cohort;; eGFR = estimated glomerular filtration rate; GFR = glomerular filtration rate; HIV = human immunodeficiency virus; IQR = interquartile range; MDRD = Modification of Diet in Renal Disease; mGFR = measured glomerular filtration rate; P_10_ = percent of eGFR values within 10% of mGFR values; P15 = percent of eGFR values with 15% of mGFR values; P_20_ = percent of eGFR values within 20% of mGFR values; P_30_ = percent of eGFR values within 30% of mGFR values; RMSE = root mean square error

Of the 12 studies analyzed for the review that compared eGFR equations with and without race adjustment to mGFR, 7 (58.3%) were cross-sectional [10, 28–33], 3 (25.0%) were retrospective [29, 33, 34], and 1 (8.3%) used pooled data from previous studies [10]; f4 (33.3%) were prospective [35–38].

Participant clinical characteristics varied; 3 studies (25.0%) focused on participants with renal dysfunction [30, 36, 37] and 5 (41.6%) included a mixed population, including patients with and without kidney disease [10, 28–29, 33, 38]. Participants with sickle cell disease [35] and HIV [31–32, 34] were also represented.

All studies evaluating CKD-EPI used the 2009 creatinine-based equation (CKD-EPI_Cr_) [5]. Three of the studies that evaluated CKD-EPI_Cr_ equations [30, 31, 34] also evaluated CKD-EPI_Cr-Cys_ equations [39], with and without race correction (see S2 File). All studies evaluating MDRD used the 4-variable equation with a constant of 175 [3]. Measured GFR was assessed using iohexol in 4 studies [28, 32, 34, 35], ^51^Cr-EDTA in 4 studies [30, 31, 33, 36], ^99m^Tc-DTPA in 2 studies [29, 38], and iothalamate in 2 studies [10, 37].

The geographic distribution of the studies varied. Three studies (25.0%) evaluated eGFR in the U.S. [10, 34, 37]. The U.S. studies included a retrospective validation study of HIV-positive and -negative individuals, which evaluated the bias and P_30_ of the CKD-EPI_Cr_ equation [33], a cross-sectional validation study of patients with and without CKD, which evaluated the bias and root mean square error (RMSE) of the CKD-EPI_Cr_ equation [10], and a prospective cohort study using data from the Chronic Renal Insufficiency Cohort (CRIC) study, which evaluated the bias of CKD-EPI_Cr_ [37]. Six studies (50.0%) evaluated eGFR in African countries: South Africa [29, 31, 36, 38], the Ivory Coast [28], the Democratic Republic of the Congo [28], and Kenya [32]. One study (8.3%) was conducted in France [35], 1 study (8.3%) in the United Kingdom (UK) [33], and 1 (8.3%) was conducted in Brazil [30].

Table 2 presents the bias, accuracy, and precision performance results of the eGFR equations.

**Table 2:** Studies evaluating bias, accuracy, and precision, with and without race adjustment.

			Bias^a^		Accuracy		Precision	
Reference and eGFR equation	Population; #Black participants	mGFR	With race adjustment	Without race adjustment	With race adjustment	Without race adjustment	With race adjustment	Without race adjustment
***CKD-EPI***_***Cr***_ ***equation***								
Arlet, 2012 [35] CKD-EPI_Cr_	Patients with sickle cell disease; n = 64	Iohexol	Mean bias (95% CI): 30.2 (25.8–35.2) Median bias (IQR): 30.5 (16.5–44.3)	Mean bias (95% CI): 10.7 (5.8–15.7) Median bias (IQR): 12.8 (-0.7–24.8)	NR	NR	NR	NR
Atta, 2021 [34] CKD-EPI_Cr_	Individuals who are HIV-positive and HIV-negative; n = 327	Iohexol	HIV positive: Bias^a^ (95% CI): 9.1 (7.2, 11.0) HIV negative: Bias (95% CI): 5.1 (2.5, 7.7)	HIV positive: Bias (95% CI): -3.9 (-5.8, -2.1) HIV negative: Bias (95% CI): -8.2 (-10.7, -5.7)	HIV positive: P30, % (95% CI): 78.4 (76–81) HIV negative: P30, % (95% CI): 87.9 (85–91)	HIV positive: P30, % (95% CI): 86.3 (84–89) HIV negative: P30, % (95% CI): 88.1 (85–91)	NR	NR
Bukabau, 2019 [28] CKD-EPI_Cr_	Mix: individuals with and without CKD; n = 494	Iohexol	Absolute bias (95% CI): 13.3 (11.4 to 15.2)	Absolute bias (95% CI): 0.0 (-1.6 to 1.6)	P30,% (95% CI): 64.6 (60.3 to 68.8)	P30,% (95% CI): 77.7 (74.1 to 81.4)	Median precision, SD: 21.3	Median precision, SD: 18.1
Gama, 2021 [33] CKD-EPI_Cr_	Patients at a large tertiary hospital; n = 266	^51^Cr-EDTA	Median absolute bias:^a^ 20.0 Mean absolute bias: 20.3 Mean percentage bias: 29.5	Median absolute bias: 7.0 Mean absolute bias: 6.7 Mean percentage bias:11.8	P30, %: 56.4	P30, %: 77.1	Precision^b^: 21.8	Precision: 19.4
Holness, 2020 [38] CKD-EPI_Cr_	Patients with CKD, potential kidney donors, healthy volunteers; n = 80	^99m^Tc-DTPA	Median bias (95% CI): 20.3 (14.6–24.0)	Median bias (95% CI): 7.9 (5.4–11.5)	P30, % (95% CI): 47.5 (36.2–59.0) P20, % (95% CI): 31.3 (21.4–42.6)	P30, % (95% CI): 72.5 (61.4–81.9) P20, % (95% CI): 55.0 (43.5–66.2)	Precision^c^ IQR of the differences: 28.1 RMSE^d^: 0.347	Precision IQR of the differences: 20.2 RMSE: 0.257
Levey, 2020 [10] CKD-EPI_Cr_	Patients with and without CKD; n = 2601	Iothalamate	Median bias (95% CI):^a^ 0 (-0.5–0.6);	Median bias (95% CI): -4.0 (-4.5- -3.5)	NR	NR	RMSE^e^ (95% CI): 0.243 (0.232–0.254)	RMSE (95% CI): 0.258 (0.248–0.268)
Moodley, 2018 [29] CKD-EPI_Cr_	Inpatients and outpatients; mix of conditions; n = 188	^99m^Tc-DTPA	Mean bias,^a^ %: Female: 31.5 Male: 39.4	Mean bias, %: Female: 13.5 Male: 20.2	P10, %: Female: 16.8 Male: 16 P30, %: Female: 46.7; Male: 45.7	P10, %: Female: 20.6 Male: 19.8 P30, %: Female: 53.3; Male: 54.3	NR	NR
Rocha, 2020 [30] CKD-EPI_Cr_	Patients with CKD; n = 61	^51^Cr-EDTA	Absolute bias (IQR): 3.2 (−0.5–14.3)	Absolute bias (IQR): −0.5 (−8.1–5.9)	P30, % (95% CI): 67.2 (54.7–77.7)	P30, % (95% CI): 75.4 (63.2–84.6)	Median precision^f^ (IQR): 5.5 (2.0–14.3)	Median precision (IQR): 7.4 (3.0–13.3)
Seape, 2016 [31] CKD-EPI_Cr_	Patients with HIV who are ART-naïve; n = 97	^51^Cr-EDTA	Proportional bias, % (95% CI): 33.7 (25.0–42.4) Median bias (95% CI): 26.7 (20.8–32.0)	Proportional bias, % (95% CI): 15.3 (7.8–22.8) Median bias (95% CI): 10.2 (5.2–15.4)	P15, %: 24.7 P30, %: 41.2	P15, %: 35.1 P30, %: 62.9	Median precision^b^, SD, 95% limit of agreement: 43.2 (-51.1–118.4)	Median precision, SD, 95% limit of agreement: 37.3 (-57.8–88.4)
Wyatt, 2013 [32] CKD-EPI_Cr_	Individuals with HIV who are ART-naïve and ambulatory; n = 99	Iohexol (dried blood spots)	Bias ratio: 1.10	Bias ratio: 0.96	P30, %: 82 P10, %: 32	P30, %: 85 P10, %: 35	NR	NR
Zelnick, 2021 [37] CKD-EPI_Cr_	Patients with chronic renal insufficiency; n = 311 patients with iGFR of 15 to less than 45 mL/min/1.73m^2^ measured within 60 days of the CRIC study visit.	^125^I-iothalamate	Mean bias: Participants with: iGFR of 15 to <45 mL/min/1.73 m^2^ (n = 311 pts; 470 unique measurements): 3.1 (2.2 to 3.9)	Mean bias: Participants with iGFR of 15 to <45 mL/min/1.73 m^2^ (n = 311 pts; 470 unique measurements): -1.7 (-2.5 to -0.9)	NR	NR	NR	NR
***MDRD equation***								
Arlet, 2012 [35] MDRD	Patients with sickle cell disease; n = 64	Iohexol	Mean bias (95% CI): 48.7 (40.0–58.4) Median bias (IQR): 49.3 (24.7–64.8)	Mean bias (95% CI): 20.7 (12.9–28.5) Median bias (IQR): 19.9 (4.9–32.9)	NR	NR	NR	NR
Bukabau, 2019 [28] MDRD	Mix: individuals with and without CKD; n = 494	Iohexol	Absolute bias (95% CI): 8.2 (6.1 to 10.2)	Absolute bias (95% CI): -7.8 (-9.5 to -6.1)	P30,% (95% CI): 73.3 (69.4 to 77.2)	P30,% (95% CI): 76.1 (72.3 to 79.9)	Median precision, SD: 23.3	Median precision, SD: 19.4
Gama, 2021 [34] MDRD	Patients at a large tertiary hospital; n = 266	^51^Cr-EDTA	Mean absolute bias: 19.7 Median absolute bias: 16.0 Mean percentage bias: 28.1	Mean absolute bias: 2.4 Median absolute bias: 1.0 Mean percentage bias: 5.6	P30, %: 56.8	P30, %: 75.2	Precision, SD:27.1	Precision, SD: 22.8
Holness, 2020 [38] MDRD	Patients with CKD, potential kidney donors, healthy volunteers; n = 80	^99m^Tc-DTPA	Median bias, (95% CI): 15.3 (11.1–20.3)	Median bias, (95% CI): 1.6 (-0.3–7.5)	P30, %, (95% CI): 51.3 (39.9–62.6) P20, %, (95% CI): 36.3 (25.8–47.8) RMSE^g^: 0.331	P30, %, (95% CI): 80 (69.6–88.1) P20, %, (95% CI): 60 (48.4–70.8) RMSE^g^: 0.239	Median precision (IQR^h^): 25.9 Median precision (RMSE)^d^: 18.2	Median precision (IQR^h^): 15.9 Median precision (RMSE)^d^: 15.1
Seape, 2015 [31] MDRD	Patients with HIV who are ART-naïve; n = 97	^51^Cr-EDTA	Median bias (95% CI): 28.2 (20.5–36.7) Proportional bias (95% CI): 38.4 (27.5–49.3)	Median bias (95% CI): 15.0 (3.5–35.9) Proportional bias (95% CI): 14.2 (5.2–23.2)	P30, %: 43.3^i^ P15, %: 22	P30, %: 59.8 P15, %: 27.8	Median precision, SD, (95% LOA): 54.0 (-67.4–144.3)	Median precision, SD, (95% LOA): 44.6 (-73.1–101.6)
Van Deventer, 2008 [36] MDRD	Patients with established CKD or at risk of CKD; n = 100	^51^Cr-EDTA	Median bias, (95% CI): 13.1 (5.5–18.3) Median percentage bias: 27.0	Median bias, (95% CI): 1.9 (-0.8–4.5) Median percentage bias: 4.8	P30, %: 52	P30, %: 74	Median precision (IQR^c^): 25.2 Median precision (RMSE): 28.5	Median precision (IQR^c^): 16.4 Median precision (RMSE): 16.6
Wyatt, 2013 [32] MDRD	Individuals with HIV who are ART-naïve and ambulatory; n = 99	Iohexol (dried blood spots)	Bias ratio: 1.18	Bias ratio: 0.97	P30, %: 73 P10, %: 26	P30, %: 83 P10, %: 34	NR	NR

^a^Bias differences were calculated as eGFR- mGFR (units in ml/min per 1.73 m^2^); Percentage bias was calculated by study authors as (eGFR-mGFR)/eGFR.

^b^SD of the bias

^c^IQR of the difference between eGFR and mGFR

^d^RMSE of eGFR vs. mGFR regression

^e^RMSE = “square root of the mean of squared differences between mGFR and eGFR”

^f^Precision = “median and interquartile interval of the difference between estimated GFR and measured GFR”

^g^Authors calculated RMSE for both accuracy and precision

^h^Interquartile range for the difference between estimated and measured GFR

^i^This value is reported as 48.3% in the narrative results and 43.3% in Table 2 of the paper.

Abbreviations: ^51^Cr-EDTA = chromium-51 labeled ethylenediamine tetraacetic acid; ^99^mTc-DTPA = technetium-99m diethylenetriamine pentaacetic acid; ART = antiretroviral therapy; CI = confidence interval; CKD = chronic kidney disease; CKD-EPI = Chronic Kidney Disease Epidemiology Collaboration; CRIC = Chronic Renal Insufficiency Cohort; Cr = creatinine; eGFR = estimated glomerular filtration rate; GFR = glomerular filtration rate; HIV = human immunodeficiency virus; iGFR = iothalamate glomerular filtration rate; IQR = interquartile range; LOA = limits of agreement; MDRD = Modification of Diet in Renal Disease; mGFR = measured glomerular filtration rate; NR = not reported; RMSE = root mean square error; P10 = percent of eGFR values within 10% of mGFR values; P15 = percent of eGFR values with 15% of mGFR values; P20 = percent of eGFR values within 20% of mGFR values; P30 = percent of eGFR values within 30% of mGFR values; SD = standard deviation

### Bias

Of the 11 studies evaluating bias of CKD-EPI_Cr_ equations, with and without race adjustment, bias improved with removal of race adjustment in 10 studies (Table 2) [28–35, 37–38], including 2 U.S. studies [13, 37]. By contrast, a U.S. study of patients with and without CKD that used the original data set from which the CKD-EPI_Cr_ equation was derived found that bias worsened with removal of race adjustment [10].

In all 7 studies evaluating bias of the 4-variable MDRD equation with and without race adjustment, bias improved with race adjustment removal [28, 31–34, 36, 38].

### Accuracy

All 8 studies, including one from the U.S. [33], evaluating the accuracy of CKD-EPI_Cr_ equations, with and without race adjustment (Table 2), found improvement with removal of the race coefficient [28–34, 38].

In all 6 studies assessing the accuracy of MDRD equations with and without race adjustment, removal of the race coefficient improved accuracy (Table 2) [28, 31–33, 36, 38]. Across all studies reporting accuracy outcomes, irrespective of eGFR equation evaluated, all used P_30_ as an accuracy measure. Additional accuracy thresholds reported included P_20_ [38], P_15_ [31], and P_10_ [29, 32].

### Precision

Precision improved with removal of race adjustment in 4 of 6 studies evaluating the precision of CKD-EPI_Cr_, with and without race adjustment (Table 2) [28, 31, 33, 38]. Precision was evaluated via different methodologies, including RMSE—calculated as the square root of the mean squared differences of eGFR and mGFR, the interquartile interval (IQR) of the difference between eGFR and mGFR, standard deviation of bias, or the interquartile range of the eGFR and mGFR differences. In 2 studies, including a U.S. study [10] and a study in a Brazilian population [30], precision worsened with the removal of race adjustment.

Five studies evaluated precision of the 4-variable MDRD equation (Table 2) and reported improvement when race adjustment was removed [28, 31, 33, 36, 38]. Precision was measured as standard deviation of bias or the interquartile range of the eGFR and mGFR differences.

## Correlation and concordance

Overall, the findings were mixed in the 6 studies reporting correlation between CKD-EPI_Cr_ equations, with and without race adjustment, and mGFR (S4 Table) [28–30, 32, 35, 38]. Arlet and colleagues (2012) found that the difference between eGFR and mGFR decreased with increasing GFR values for CKD-EPI_Cr_ with race adjustment (r = -0.23, p = 0.06) and without race adjustment (r = -0.43, p<0.001) [35]. Bukabau et al. report increased correlation between eGFR and mGFR in a large cohort of participants from the Democratic Republic of Congo and the Ivory Coast with removal of the race coefficient compared to inclusion of the race coefficient (Lin’s Concordance Correlation Coefficient of 0.81 vs. 0.71 respectively) [28]. Rocha and colleagues found that concordance was 52.5% for African Brazilians using CKD-EPI_Cr_ with race adjustment, and 54.1% without race adjustment [30]. Moodley and colleagues [29] observed a high correlation both with and without race adjustment (R^2^ = 0.83 for females; R^2^ = 0.86 for males). Holness (2020) reports agreement between CKD-EPI_Cr_ and mGFR; with race adjustment, the 95% limits of agreement were -14.5–55.2 and without race adjustment, -17.9–35.7 [38]. However, correlation was poor in a study by Wyatt and colleagues (R^2^ = 0.23 both with and without race adjustment) [32].

In 3 studies assessing the correlation between MDRD and mGFR (S4 Table), two reported increased correlation with removal of eGFR race adjustment [28, 35]. However, Wyatt and colleagues report no difference in correlation or variance inflation when the race variable is removed from the eGFR equation [32].

### Social determinants of health and non-creatinine determinants of GFR

Only 2 studies reported social determinants of health and neither provided eGFR vs. mGFR performance outcomes (i.e., bias, accuracy, precision) by social determinants of health [34, 37]. Smoking status was reported in both studies, with Atta at al. also describing the income, education level, and insurance status of their study population (Black patients with mild to moderate CKD) [34]. Non-GFR determinants of creatinine were reported at baseline in 4 studies [32, 34, 35, 38]; however, none reported eGFR vs. mGFR performance by non-GFR determinants of creatinine.

### Quality assessment outcomes

Three studies analyzed for the review had low risk of bias for the patient selection domain (Table 3). Nine studies had unclear risk of bias for patient selection (Table 3), including 3 retrospective studies [29, 33, 34] and 1 study evaluating outcomes based on multiple measurements for some patients [34]. All 12 studies analyzed for the review were rated as having unclear risk of bias for the index test and reference standard domains due to a lack of information about whether the tests were interpreted without knowledge of each other. Regarding flow and timing, 6 studies had low risk of bias [28–30, 34, 36, 38], and the remaining had unclear bias. Applicability concerns were low for most studies and domains, except for patient selection in 1 study [38]. See S5 Table for quality assessment ratings of the 22 studies not included in the narrative synthesis.

**Table 3:** QUADAS-2 risk of bias and applicability assessment of studies included in the systematic review synthesis.

First Author, Year	Patient Selection					Index Test				Reference Standard				Flow and Timing				
	Bias				Applicability concerns	Bias			Applicability concerns	Bias			Applicability concerns	Bias				
	Signaling questions			Risk of bias		Signaling questions		Risk of bias		Signaling questions		Risk of bias		Signaling questions				Risk of bias
	Was a consecutive or random sample of patients enrolled? (1a.1)	Was a case-control design avoided? (1a.2)	Did the study avoid inappropriate exclusions? (1a.3)	Could the selection of patients have introduced bias? (1a.4)	Is there concern that the included patients do not match the review question? (1b.1)	Were the index test(s) results interpreted without knowledge of the results of the reference standard? (2a.1)	If a threshold was used, was it pre-specified? (2a.2)	Could the conduct or interpretation of the test(s) have introduced bias? (2a.3)	Is there concern the index test(s), its conduct, or interpretation differ from the review question? (2b.1)	Is the reference standard likely to correctly classify the target condition? (3a.1)	Were the reference standard results interpreted without knowledge of the results of the index test? (3a.2)	Could the reference standard, its conduct or interpretation have introduced bias? (3a.3)	Are there concerns that the target condition as defined by the reference standard does not match the review question? (3b.1)	Was there an appropriate interval between index test(s) and reference standard? (4a.1)	Did all patients receive a reference standard? (4a.2)	Did patients receive the same reference standard? (4a.3)	Were all patients included in the analysis? (4a.4)	Could the patient flow have introduced bias? (4a.5)
Arlet, 2012 [35]	Y	Y	Y	**L**	**L**	U	N/A	**U**	**L**	Y	U	**U**	**L**	U	Y	Y	**Y**	**U**
Atta, 2021 [34]	U	Y	Y	**U**	**L**	U	Y	**U**	**L**	Y	U	**U**	**L**	Y	Y	Y	**Y**	**L**
Bukabau, 2019 [28]	U	Y	Y	**U**	**L**	U	Y	**U**	**L**	Y	U	**U**	**L**	Y	Y	Y	**Y**	**L**
Gama, 2021 [33]	U	Y	Y	**U**	**L**	U	Y	**U**	**L**	Y	U	**U**	**L**	N	Y	Y	**Y**	**U**
Holness, 2020 [38]	U	Y	Y	**U**	**H**	U	Y	**U**	**L**	Y	U	**U**	**L**	Y	Y	Y	**Y**	**L**
Levey, 2020 [10]	N	Y	Y	**U**	**L**	U	N/A	**U**	**L**	Y	U	**U**	**L**	U	Y	Y	**Y**	**U**
Moodley, 2018 [29]	U	Y	Y	**U**	**L**	U	Y	**U**	**L**	Y	U	**U**	**L**	Y	Y	Y	**Y**	**L**
Rocha, 2020 [30]	U	Y	Y	**U**	**L**	U	Y	**U**	**L**	Y	U	**U**	**L**	Y	Y	Y	**Y**	**L**
Seape, 2016 [31]	U	Y	Y	**U**	**L**	U	Y	**U**	**L**	Y	U	**U**	**L**	U	Y	Y	**U**	**U**
Van Deventer, 2008 [36]	U	Y	Y	**U**	**L**	U	Y	**U**	**L**	Y	U	**U**	**L**	Y	Y	Y	**Y**	**L**
Wyatt, 2013 [32]	Y	Y	Y	**L**	**L**	U	N/A	**U**	**L**	U	U	**U**	**L**	U	Y	N	**N**	**U**
Zelnick, 2021 [37]	Y	Y	Y	**L**	**L**	U	Y	**U**	**L**	Y	U	**U**	**L**	U	Y	Y	**N**	**U**

Responses options are as follows: signaling questions (Y, N, U); risk of bias (H, L, U); applicability (H, L, U).

Abbreviations: H = High risk; L = Low risk; N = No; U = Unclear; Y = Yes, N/A = Not applicable

## Discussion

Previous efforts revealed the need for a comprehensive, systematic review of international scope to inform the use of eGFR estimation in Black patients [40], and our study is among the first systematic reviews to assess worldwide evidence related to adjustment for Black race in eGFR equations. The most commonly reported performance metrics identified from our systematic review were bias, precision, and accuracy, which improved in the majority of studies when race was removed from eGFR equations. Concordance and correlation were less often evaluated and seldom assessed in a standardized way, making it difficult to draw conclusions. Results from the QUADAS-2 quality assessment indicated that while there was low concern regarding the applicability of the included studies for the overall systematic review question, risk of study bias was difficult to assess due to inadequate reporting and variations in study methodology and design.

Recently, new recommendations have been put forth by NKF-ASN and the National Institute for Health and Care Excellence (NICE) to remove race adjustment from eGFR equations [20, 21, 41, 42]. The NKF-ASN task force recommended a new CKD-EPI_Cr_ equation refit without a race adjustment for U.S. adults [20, 21]. We did not include the study reporting the new CKD-EPI_Cr_ equation [21] in our systematic review, since it was published outside of our pre-established date parameters. While the NKF-ASN task force recommended use of the new CKD-EPI_Cr_ equation refit without race, they noted that accuracy may improve with use of equations combining filtration markers. Inker and colleagues (2021) found that use of a new CKD-EPI equation incorporating both creatinine and cystatin C, without race adjustment (new CKD-EPI_Cr-Cys_), was more accurate than the new CKD-EPI_Cr_ equation, which underestimated mGFR in Black adults and overestimated mGFR in non-Black adults in the U.S. [21]. Future validation studies are needed to assess the performance of the new CKD-EPI equations in other countries and across a variety of clinical settings.

Our study has several limitations. We only included studies that compared the performance of eGFR equations both with and without race adjustment. We did not evaluate differences in performance between the multiple versions of the CKD-EPI and MDRD equations, thought less clinically relevant especially given recent guidelines. Measured GFR was not assessed consistently between studies. Many of the included studies did not assess the statistical significance of outcome measures when race adjustment was removed. None of the included studies assessed the impact of social determinants of health and non-creatinine determinants of GFR. Many studies did not report how race was determined; since race is inconsistently defined across populations [43], this contributes to heterogeneity in the findings. Risk of overall study bias was unclear for many studies due to inadequate reporting and variations in study methodology. A further limitation is the potential risk of publication bias; however, we minimized this risk by conducting a comprehensive literature review that spanned across multiple databases and included grey literature sources.

Our study focused on Black participants rather than mixed populations. While we recognize the value in studies with mixed populations, we chose to focus on studies with Black participants in order to optimally assess the study’s main objective of determining how well eGFR with and without race adjustment estimates mGFR in Black adults across the world. Given the paucity of data on eGFR in Black people in the U.S., and the historical limited participation of Black people in clinical trials including kidney-focused trials [44], the focus of this review on Black participants is an added strength of our study.

Limitations in eGFR due to variation in non-GFR determinants of serum creatinine could be relevant in our systematic review, which included studies with heterogenous populations. In studies of sicker patient populations, eGFR_Cr_ likely overestimates kidney function, and removal of the race coefficient can bring kidney function closer to the expected value. However, such findings are coincidental and do not provide additional evidence to support that eGFR equations without the race coefficient outperform eGFR equations with the race coefficient. The sub-optimal accuracy measures observed in many of the studies included in our systematic review support the need for continued efforts to improve the accuracy of eGFR within and outside the U.S. More modern and elaborate approaches are gradually replacing existing simplistic methods used in MDRD and CKD-EPI equations. Examples include Q-values in the new EKFC-equation [45] and creatinine growth curves [46]. Widespread use of these elaborate methods is currently limited by a paucity of external validation studies.

This novel, in-depth and worldwide systematic review, provides the highest level of evidence against race adjustment when estimating GFR [20, 21, 41, 42]. Race is an inappropriate proxy for genetics all over the world, and efforts to eliminate its use in estimating GFR should be global. In addition to tracking the performance of the newly recommended race-free eGFR equations, future research should prioritize health equity [47, 48] by assessing the impact of social determinants of racial disparities in kidney disease on GFR estimates and ensuring enrollment of diverse participants across the world in validation trials of GFR equations [47, 48].

## Supporting information

S1 File Search strategy and processes details. (DOCX)

S2 File Studies evaluating CKD-EPI_Cr-Cys_ equations with and without race adjustment. (DOCX)

S1 Table Systematic review protocol. (DOCX)

S2 Table PRISMA checklist. (DOCX)

S3 Table Summary characteristics of studies not included in the systematic review synthesis. (DOCX)

S4 Table Correlation or concordance tables for CKD-EPI_Cr_ and MDRD studies with and without race adjustment. (DOCX)

S5 Table QUADAS-2 risk of bias and applicability assessment of studies not included in the systematic review synthesis. (DOCX)
