# Supplementary material for: Systematic review of international studies evaluating MDRD and CKD-EPI estimated glomerular filtration rate (eGFR) equations in Black adults
Source: PLoS One. 2022 Oct 18;17(10):e0276252. doi: 10.1371/journal.pone.0276252 (PMC9578594; doi:10.1371/journal.pone.0276252)
Supplement: S1 File — (DOCX) [file pone.0276252.s001.docx]

**S3 Tables and Text. Search strategy and processes details**

**PubMed/MEDLINE Search Strategy**

| **Search** | **Strategy** |
| --- | --- |
| 1 | ("Glomerular Filtration Rate"[Mesh] OR "glomerular filtration rate"[tiab] OR "glomerular filtration rates"[tiab] OR GFR[tiab] OR kidney function[tiab] OR renal function[tiab] OR kidney function tests[mh:noexp]) AND (estimated[tiab] OR estimate[tiab] OR estimates[tiab] OR calculation[tiab] OR calculated[tiab] OR calculate[tiab] OR algorithm*[tiab] OR equat*[tiab] OR formula*[tiab] OR correction*[tiab] OR adjustment*[tiab] OR eGFR[tiab] OR “Modification of Diet in Renal Disease”[tiab] OR MDRD[tiab] OR “Chronic Kidney Disease Epidemiology”[tiab] OR “Chronic Kidney Disease Epidemiological”[tiab] OR CKD-EPI[tiab] OR “African American Study of Kidney Disease”[tiab] OR AASK[tiab]) |
| 2 | (African Continental Ancestry Group[mh] OR Ethnic Groups[mh] OR race[tiab] OR racial[tiab] OR Race Factors[mh] OR minority[tiab] OR minority health[mh] OR minority groups[mh] OR minorities[tiab] OR ethnicity[tiab] OR ethnic[tiab] OR ethnicities[tiab] OR racial[tiab] OR Africa[mh] OR Africa*[tiab] OR Afro*[tiab] OR African American[tiab] OR African Americans[tiab] OR Black American[tiab] OR Black Americans[tiab] OR Black*[tiab] OR “racial and ethnic groups”[tiab] OR ethnic differences[tiab] OR non-white[tiab] OR non-Caucasian OR “race/ethnicity” [tiab] OR Multi-ethnic[tiab] OR African ancestry[tiab] OR Black participants[tiab] OR Black study participants[tiab] OR Black subjects[tiab] OR Black adults[tiab] OR Black men[tiab] OR Black women[tiab] OR Black individuals[tiab] OR diverse[tiab] OR “people of color”[tiab] OR “people of colour”[tiab] OR “persons of color”[tiab] OR “persons of colour”[tiab] OR POC[tiab] OR WOC[tiab] OR “women of color”[tiab] OR MOC[tiab] OR “men of color”[tiab] OR BIPOC[tiab] OR BAME[tiab] OR BME[tiab] OR “minority ethnic”[tiab] OR biracial[tiab] OR multiracial[tiab] OR Angola[tiab] OR Benin[tiab] OR Botswana[tiab] OR Burkina Faso[tiab] OR Burundi[tiab] OR Cameroon[tiab] OR Cape Verde[tiab] OR Central African Republic[tiab] OR Chad[tiab] OR Comoros[tiab] OR Congo Democratic Republic[tiab] OR Cote d'Ivoire[tiab] OR Ivory Coast[tiab] OR Djibouti[tiab] OR Equatorial Guinea[tiab] OR Eritrea[tiab] OR Ethiopia[tiab] OR Gabon[tiab] OR Gambia[tiab] OR Ghana[tiab] OR Guinea[tiab] OR Guinea Bissau[tiab] OR Kenya[tiab] OR Lesotho[tiab] OR Liberia[tiab] OR Madagascar[tiab] OR Malawi[tiab] OR Mali[tiab] OR Mauritania[tiab] OR Mauritius[tiab] OR Mozambique[tiab] OR Namibia[tiab] OR Niger[tiab] OR Nigeria[tiab] OR Rwanda[tiab] OR “Sao Tome and Principe”[tiab] OR Senegal[tiab] OR Seychelles[tiab] OR Sierra Leone[tiab] OR Somalia[tiab] OR South Africa[tiab] OR South Sudan[tiab] OR Sudan[tiab] OR Swaziland[tiab] OR Tanzania[tiab] OR Togo[tiab] OR Uganda[tiab] OR Zambia[tiab] OR Zimbabwe[tiab] OR sub-Saharan[tiab] OR Angolan[tiab] OR Beninese[tiab] OR Batswana[tiab] OR Motswana[tiab] OR Burkinabe[tiab] OR Burkinese[tiab] OR Burundian[tiab] OR Cameroonian[tiab] OR “Cape Verdean”[tiab] OR “Cabo Verdean”[tiab] OR “Central African”[tiab] OR Chadian[tiab] OR Comorian[tiab] OR Congolese[tiab] OR Ivorian[tiab] OR Dijiboutian[tiab] OR Equatoguinean[tiab] OR Eritrean[tiab] OR Ethiopian[tiab] OR Gabonian[tiab] OR Ghanaian[tiab] OR Guinean[tiab] OR Kenyan[tiab] OR Mosotho[tiab] OR Basotho[tiab] OR Lesothan[tiab] OR Lesothonian[tiab] OR Liberian[tiab] OR Malagasy[tiab] OR Malawian[tiab] OR Malian[tiab] OR Mauritanian[tiab] OR Mozambican[tiab] OR Namibian[tiab] OR Nigerian[tiab] OR Rwandan[tiab] OR Rwandese[tiab] OR Senegalese[tiab] OR Seychellois[tiab] OR Seychelloise[tiab] OR Seselwa[tiab] OR “Sierra Leonean”[tiab] OR Somali[tiab] OR “South African”[tiab] OR “South Sudanese”[tiab] OR Swati[tiab] OR Swazi[tiab] OR Tanzanian[tiab] OR Togolese[tiab] OR Ugandan[tiab] OR Zambian[tiab] OR Zimbabwan[tiab] OR Zimbo[tiab] OR West Indies[mh] OR “West Indies”[tiab] OR “Antigua and Barbuda”[tiab] OR Antiguan Barbudan[tiab] OR Bahamas[tiab] OR Bahamian[tiab] OR Barbados[tiab] OR Barbadian[tiab] OR “British Virgin Islands”[tiab] OR Virgin Islanders[tiab] OR Cuba[tiab] OR Cuban[tiab] OR Dominica[tiab] OR Dominican[tiab] OR Grenada[tiab] OR Grenadian[tiab] OR Guadeloupe[tiab] OR Guadeloupean[tiab] OR Martinique[tiab] OR Martinican[tiab] OR Puerto Rico[tiab] OR Puerto Rican[tiab] OR “Saint Kitts and Nevis”[tiab] OR Kittitian[tiab] OR Nevisian[tiab] OR Saint Lucia[tiab] OR Saint Lucian[tiab] OR “Saint Vincent and the Grenadines”[tiab] OR Saint Vincentian[tiab] OR “Trinidad and Tobago”[tiab] OR Trinidadian[tiab] OR Tobagonian[tiab] OR “United States Virgin Islands”[tiab] OR “US Virgin Islands”[tiab] OR Jamaica[tiab] OR Jamaican[tiab] OR Haiti[tiab] OR Haitian[tiab] OR Dominican Republic[tiab] OR United Kingdom[mh] OR United Kingdom[tiab] OR Great Britain[tiab] OR Brazil[mh] OR Brazil[tiab] OR Brazilian[tiab] OR Brasilian[tiab] OR Mexico[mh] OR Mexico[tiab] OR Mexican[tiab] OR Venezuela[mh] OR Venezuela[tiab] OR Venezuelan[tiab] OR Colombia[mh] OR Colombia[tiab] OR Colombian[tiab] OR Canada[mh] OR Canada[tiab] OR Canadian[tiab]) |
| 3 | #1 AND #2 |
| 4 | #3 NOT (news[pt] OR historical article[pt]) |
| 5 | #4 AND (English[la] OR French[la] OR Spanish[la] OR Portuguese[la]) |
| 6^a^ | #5 AND (1999:2020[pdat]) |
| 7^b^ | #5 AND (2020:2021[pdat] OR 2020:2021[edat] OR 2020:2021[crdt]) |

^a^Search strategy executed on October 23, 2020

^b^Search strategy re-executed on May 6, 2021

Abbreviations: * = PubMed truncation symbol; [crdat]=Create Date; [edat]=Entry Date; [la]=Language; [MeSH]=Medical Subject Headings; [mh]=Medical Subject Headings; noexp=No Explosion; [pdat]=Publication Date; [pt]=Publication Type; [tiab]=Title/Abstract

**Web of Science Search Strategy.** *Databases searched: Web of Science Core Collection, BIOSIS Citation Index, MEDLINE, SciELO Citation Index*

| **Search** | **Strategy** |
| --- | --- |
| #1 | TS=(Glomerular Filtration Rate* NEAR (estimated OR estimate OR estimates OR calculation OR calculated OR calculate OR algorithm* OR equat* OR formula* OR correction* OR adjustment* OR eGFR OR MDRD OR CKD-EPI OR AASK OR “Modification of Diet in Renal Disease” OR “Chronic Kidney Disease Epidemiology” OR “Chronic Kidney Disease Epidemiological” OR “African American Study of Kidney Disease”)) OR TS=(GFR NEAR (estimated OR estimate OR estimates OR calculation OR calculated OR calculate OR algorithm* OR equat* OR formula* OR correction* OR adjustment* OR eGFR OR MDRD OR CKD-EPI OR AASK OR “Modification of Diet in Renal Disease” OR “Chronic Kidney Disease Epidemiology” OR “Chronic Kidney Disease Epidemiological” OR “African American Study of Kidney Disease”)) OR TS=(“kidney function” NEAR (estimated OR estimate OR estimates OR calculation OR calculated OR calculate OR algorithm* OR equat* OR formula* OR correction* OR adjustment* OR eGFR OR MDRD OR CKD-EPI OR AASK OR “Modification of Diet in Renal Disease” OR “Chronic Kidney Disease Epidemiology” OR “Chronic Kidney Disease Epidemiological” OR “African American Study of Kidney Disease”)) OR TS=(“renal function” NEAR (estimated OR estimate OR estimates OR calculation OR calculated OR calculate OR algorithm* OR equat* OR formula* OR correction* OR adjustment* OR eGFR OR MDRD OR CKD-EPI OR AASK OR “Modification of Diet in Renal Disease” OR “Chronic Kidney Disease Epidemiology” OR “Chronic Kidney Disease Epidemiological” OR “African American Study of Kidney Disease”)) |
| #2 | TS=(race OR racial OR minority OR minorities OR ethnicity OR ethnic OR ethnicities OR Africa* OR Afro* OR African American* OR Black American* OR Black* OR “non-white” OR “non-Caucasian” OR “race/ethnicity” OR Multi-ethnic OR “African ancestry” OR “Black participants” OR “Black study participants” OR “Black subjects” OR “Black adults” OR “Black men” OR “Black women” OR “Black individuals” OR diverse OR “people of color” OR “people of colour” OR “persons of color” OR “persons of colour” OR POC OR WOC OR “women of color” OR MOC OR “men of color” OR BIPOC OR BAME OR BME OR “minority ethnic” OR biracial OR multiracial OR Angola OR Benin OR Botswana OR “Burkina Faso” OR Burundi OR Cameroon OR “Cape Verde” OR “Central African Republic” OR Chad OR Comoros OR “Congo Democratic Republic” OR “Cote d'Ivoire” OR “Ivory Coast” OR Djibouti OR “Equatorial Guinea” OR Eritrea OR Ethiopia OR Gabon OR Gambia OR Ghana OR Guinea OR “Guinea Bissau” OR Kenya OR Lesotho OR Liberia OR Madagascar OR Malawi OR Mali OR Mauritania OR Mauritius OR Mozambique OR Namibia OR Niger OR Nigeria OR Rwanda OR “Sao Tome and Principe” OR Senegal OR Seychelles OR “Sierra Leone” OR Somalia OR “South Africa” OR “South Sudan” OR Sudan OR Swaziland OR Tanzania OR Togo OR Uganda OR Zambia OR Zimbabwe OR “sub-Saharan” OR Angolan OR Beninese OR Batswana OR Motswana OR Burkinabe OR Burkinese OR Burundian OR Cameroonian OR “Cape Verdean” OR “Cabo Verdean” OR “Central African” OR Chadian OR Comorian OR Congolese OR Ivorian OR Dijiboutian OR Equatoguinean OR Eritrean OR Ethiopian OR Gabonian OR Ghanaian OR Guinean OR Kenyan OR Mosotho OR Basotho OR Lesothan OR Lesothonian OR Liberian OR Malagasy OR Malawian OR Malian OR Mauritanian OR Mozambican OR Namibian OR Nigerian OR Rwandan OR Rwandese OR Senegalese OR Seychellois OR Seychelloise OR Seselwa OR “Sierra Leonean” OR Somali OR “South African” OR “South Sudanese” OR Swati OR Swazi OR Tanzanian OR Togolese OR Ugandan OR Zambian OR Zimbabwan OR Zimbo OR “West Indies” OR “Antigua and Barbuda” OR “Antiguan Barbudan” OR Bahamas OR Bahamian OR Barbados OR Barbadian OR “British Virgin Islands” OR “Virgin Islanders” OR Cuba OR Cuban OR Dominica OR Dominican OR Grenada OR Grenadian OR Guadeloupe OR Guadeloupean OR Martinique OR Martinican OR “Puerto Rico” OR “Puerto Rican” OR “Saint Kitts and Nevis” OR Kittitian OR Nevisian OR “Saint Lucia” OR “Saint Lucian” OR “Saint Vincent and the Grenadines” OR “Saint Vincentian” OR “Trinidad and Tobago” OR Trinidadian OR Tobagonian OR “United States Virgin Islands” OR “US Virgin Islands” OR Jamaica OR Jamaican OR Haiti OR Haitian OR “Dominican Republic” OR “United Kingdom” OR “Great Britain” OR Brazil OR Brazilian OR Mexico Mexican OR Venezuela OR Venezuelan OR Colombia OR Colombian OR Canada OR Canadian) |
| #3 | #1 AND #2 |
| #4 | Exclude document types “News,” “Book,” “Reference Material” |
| #5 | Limit language to English, French, Spanish, Portuguese, or Unspecified |
| #6^a^ | Filter publication date to 1999 - 2020 |
| #7^b^ | #5, filter publication date to 2020 – 2021 |

^a^Search strategy executed on October 23, 2020

^b^Search strategy re-executed on May 6, 2021

Abbreviations: * = Web of Science truncation symbol; TS=Topic

**EMBASE Search Strategy (Ovid** **Platform)**

| **Search** | **Strategy** |
| --- | --- |
| #1 | estimated glomerular filtration rate/ or ((exp glomerulus filtration rate/ or glomerular filtration rate.tw. or glomerulus filtration rate.tw. or GFR.tw. or kidney function.tw. or renal function.tw. or exp kidney function/) adj5 (*calculation/ or estimated.tw. or estimate.tw. or estimates.tw. or calculation.tw. or calculated.tw. or calculate.tw. or algorithm*.tw. or equat*.tw. or formula*.tw. or correction*.tw. or adjustment*.tw. or eGFR.tw. or MDRD.tw. or CKD-EPI.tw. or AASK.tw. or "Modification of Diet in Renal Disease".tw. or "Chronic Kidney Disease Epidemiology".tw. or "Chronic Kidney Disease Epidemiological".tw. or "African American Study of Kidney Disease".tw.)) |
| #2 | exp Black person/ or exp African/ or exp Africa/ or exp "ethnic or racial aspects"/ or minority group/ or minority health/ or race.tw. or racial.tw. or minority.tw. or minorities.tw. or ethnicity.tw. or ethnic.tw. or ethnicities.tw. or Africa*.tw or Afro*.tw. or African American.tw. or African Americans.tw. or Black American.tw. or Black Americans.tw. or Black*.tw. or ethnic differences.tw. or non-white.tw. or non-Caucasian.tw. or Multi-ethnic.tw. or people of color.tw. or people of colour.tw. or persons of color.tw. or persons of colour.tw. or POC.tw. or WOC.tw. or women of color.tw. or MOC.tw. or men of color.tw. or BIPOC.tw. or BAME.tw. or BME.tw. or minority ethnic.tw. or biracial.tw. OR multiracial.tw. or African ancestry.tw. or Black participants.tw. or Black study participants.tw. or Black subjects.tw. or Black adults.tw. or Black men.tw. or Black women.tw. or Black individuals.tw. or diverse.tw. or Angola.tw. or Benin.tw. or Botswana.tw. or Burkina Faso.tw. or Burundi.tw. or Cameroon.tw. or Cape Verde.tw. or Central African Republic.tw. or Chad.tw. or Comoros.tw. or Congo Democratic Republic.tw. or Cote d'Ivoire.tw. or Ivory Coast.tw. or Djibouti.tw. or Equatorial Guinea.tw. or Eritrea.tw. or Ethiopia.tw. or Gabon.tw. or Gambia.tw. or Ghana.tw. OR Guinea.tw. or Guinea Bissau.tw. or Kenya.tw. or Lesotho.tw. or Liberia.tw. or Madagascar.tw. or Malawi.tw. or Mali.tw. or Mauritania.tw. or Mauritius.tw. or Mozambique.tw. or Namibia.tw. or Niger.tw. or Nigeria.tw. or Rwanda.tw. or Sao Tome.tw. or Principe.tw. or Senegal.tw. or Seychelles.tw. or Sierra Leone.tw. or Somalia.tw. or South Africa.tw. or South Sudan.tw. or Sudan.tw. or Swaziland.tw. or Tanzania.tw. or Togo.tw. or Uganda.tw. or Zambia.tw. or Zimbabwe.tw. or sub-Saharan Africa.tw. or Angolan.tw. or Beninese.tw. or Batswana.tw. or Motswana.tw. or Burkinabe.tw. or Burkinese.tw. or Burundian.tw. or Cameroonian.tw. or Cape Verdean.tw. or Cabo Verdean.tw. or Central African.tw. or Chadian.tw. or Comorian.tw. or Congolese.tw. or Ivorian.tw. or Dijiboutian.tw. or Equatoguinean.tw. or Eritrean.tw. or Ethiopian.tw. or Gabonian.tw. or Ghanaian.tw. or Guinean.tw. or Kenyan.tw. or Mosotho.tw. or Basotho.tw. or Lesothan.tw. or Lesothonian.tw. or Liberian.tw. or Malagasy.tw. or Malawian.tw. or Malian.tw. or Mauritanian.tw. or Mozambican.tw. or Namibian.tw. or Nigerian.tw. or Rwandan.tw. or Rwandese.tw. or Senegalese.tw. or Seychellois.tw. or Seychelloise.tw. or Seselwa.tw. or Sierra Leonean.tw. or Somali.tw. or South African.tw. or South Sudanese.tw. or Swati.tw. or Swazi.tw. or Tanzanian.tw. or Togolese.tw. or Ugandan.tw. or Zambian.tw. or Zimbabwan.tw. or Zimbo.tw. or exp Caribbean Islands/ or exp "Caribbean (person)"/ or West Indies.tw. or (Antigua.tw. and Barbuda.tw.) or Antiguan Barbudan.tw. or Bahamas.tw. or Bahamian.tw. OR Barbados.tw. or Barbadian.tw. or British Virgin Islands.tw. or Virgin Islanders.tw. or Cuba.tw. or Cuban.tw. or Dominica.tw. or Dominican.tw. or Grenada.tw. or Grenadian.tw. or Guadeloupe.tw. or Guadeloupean.tw. or Martinique.tw. or Martinican.tw. or Puerto Rico.tw. or Puerto Rican.tw. or (Saint Kitts.tw. and Nevis.tw.) or Kittitian.tw. or Nevisian.tw. or Saint Lucia.tw. or Saint Lucian.tw. or (Saint Vincent.tw. and Grenadines.tw.) or Saint Vincentian.tw. or (Trinidad.tw. and Tobago.tw.) or Trinidadian.tw. or Tobagonian.tw. or United States Virgin Islands.tw. or US Virgin Islands.tw. or Jamaica.tw. or Jamaican.tw. or Haiti.tw. or Haitian.tw. or Dominican Republic.tw. or exp United Kingdom/ or United Kingdom.tw. or Great Britain.tw. or exp Brazil/ or Brazil.tw. or exp Brazilian/ or Brazilian.tw. or exp Mexico/ or exp Mexican/ or Mexico.tw. or Mexican.tw. or exp Venezuela/ or Venezuela.tw. or Venezuelan.tw. or exp Venezuelan/ or exp Colombia/ or Colombian.tw. or exp Colombian/ or exp Canada/ or Canada.tw. or Canadian.tw. or exp Canadian/ |
| #3 | 1 and 2 |
| #4 | Limit 3 to (English or French or Portuguese or Spanish or no language specified) |
| #5^a^ | Limit 4 to yr="1999 -Current" |
| #6^b^ | Limit 4 to yr="2020-2021" |

^a^Search strategy executed on October 23, 2020

^b^Search strategy re-executed on May 6, 2021

Abbreviations: * = Ovid truncation symbol; adj=Adjacent; exp=Explode; tw=Text Word; yr=Year of Publication

**ScienceDirect Search Strategies**

| **Search** | **Strategy** |
| --- | --- |
| ***ScienceDirect Search Strategy 1*** | |
| #1 | Enter in “Title, abstract, or author-specified keywords” field: ("Glomerular Filtration Rate" OR GFR) (estimated OR calculation OR algorithm OR equation OR formula OR MDRD OR CKD-EPI OR eGFR) |
| #2 | Enter in “Find articles with these terms” field: (race OR racial OR ethnicity OR minority OR Africa OR African OR "African American" OR Black OR “Non-white”) |
| #3 | #1 AND #2 |
| #4 | Filter #3 to exclude the following publication types: “Encyclopedia,” “Book chapters,” “Book reviews,” “Conference info,” “Data articles,” “Examinations,” “News,” “Patent reports,” “Product reviews,” “Software publications,” and “Video articles” |
| #5^a^ | #4, filter publication date to 1999 - 2020 |
| #6^a^ | #4, filter publication date to 2020-2021 |
| ***ScienceDirect Search Strategy 2*** | |
| #7 | Enter in “Title, abstract, or author-specified keywords” field: ("Glomerular Filtration Rate" OR GFR) (AASK OR “Modification of Diet in Renal Disease” OR “Chronic Kidney Disease Epidemiology” OR “Chronic Kidney Disease Epidemiological” OR “African American Study of Kidney Disease”) |
| #8 | Enter in “Find articles with these terms” field: (race OR racial OR ethnicity OR minority OR Africa OR African OR "African American" OR Black OR “Non-white”) |
| #9^c^ | #7 AND #8, filtered to exclude publication types listed in #4 and to limit publication date to 1999 – 2020 |
| #10^d^ | #7 AND #8, filtered to exclude publication types listed in #4 and to limit publication date to 2020-2021 |

^a^Search strategy 1 executed on October 23, 2020

^b^Search strategy 1 re-executed on May 6, 2021

^c^Search strategy 2 executed on October 23, 2020

^d^Search strategy 2 re-executed on May 6, 2021

**Google Scholar Search Strategies**

Conducted using Publish or Perish software^1^

| **Search** | **Strategy** |
| --- | --- |
| ***Google Scholar Search Strategy 1*** | |
| #1 | intitle:eGFR ("African American" OR Black OR African OR Africa OR race OR ethnicity OR racial OR ethnicities OR minority) -"epidermal growth factor" |
| #2 | #1, Exclude patents |
| #3 | #2, Filter publication date to 1999 – 2020 |
| #4 | #2, Filter publication date to 2020 – 2021 |
| ***Google Scholar Search Strategy 2*** | |
| #5 | intitle:"estimated glomerular filtration rate" ("African American" OR Black OR African OR Africa OR race OR ethnicity OR racial OR ethnicities OR minority) |
| #6 | #5, Exclude patents |
| #7^a^ | #6, Filter publication date to 1999 – 2020 |
| #8^b^ | #6, filter publication date to 2020-2021 |

^a^Search strategy executed on October 23, 2020

^b^Search strategy re-executed on May 6, 2021

Note: “intitle” = term must be in title

**Searching Process Additional Details**

The systematic review searched both peer-reviewed and grey literature. Search strategies applied use of controlled vocabulary, such as Medical Subject Headings (MeSH) and Emtree, as applicable, along with keywords. To enhance the population string, terms for sub-Saharan African countries and their populace were included based on a previous systematic review,^2^ supplemented by keywords used to represent the Black populations within other countries. Selection of countries to include within the search was determined by demographic data per the CIA World Factbook,^3^ known migration patterns of Black Africans, and input from team clinical experts. Of note, demonyms were required in order to capture relevant articles not referring specifically to Black participants in the abstract. Search statements were collaboratively constructed by medical information scientists through an iterative process and reviewed and refined by the systematic review team. To test search statement effectiveness, the team verified that key articles previously identified by study team clinical experts were retrieved via the search strategies.

The publication date was limited to 1999 or later for journal articles to coincide with the initial release of the Modification of Diet in Renal Disease (MDRD) formula^4^, the earliest of the estimated glomerular filtration rate (eGFR) equations evaluated for this systematic review. Language was limited to English, French, Spanish, or Portuguese, as the most common languages in the initial search results; sufficient members of the study team were fluent in these languages to conduct dual review. As the focus of the review was validation studies or other research studies comparing eGFR and mGFR, non-research publication types such as news and historical articles were excluded. No additional limits were applied. Deduplication processes were conducted using via PHP scripts run on CSV spreadsheet files.

During the abstract and/or full-text screening, the study team flagged articles for subsequent handsearching and reference checking. Information scientists manually reviewed all references cited in each flagged article and in all articles selected for final inclusion in the systematic review. When a relevant conference abstract was identified during screening process, manual searching was also conducted to identify any related full-length journal publications.

**References**

1. Harzing A. Publish or Perish; 2007. <https://harzing.com/resources/publish-or-perish>
2. Fabian J, George JA, Etheredge HR, et al. Methods and reporting of kidney function: a systematic review of studies from sub-Saharan Africa. Clin Kidney J. 2019;12(6):778-787. doi:10.1093/ckj/sfz089
3. Central Intelligence Agency. The World Factbook. Published 2020. Accessed October 23, 2020. <https://www.cia.gov/the-world-factbook/>
4. Levey AS, Bosch JP, Lewis JB, Greene T, Rogers N, Roth D. A more accurate method to estimate glomerular filtration rate from serum creatinine: a new prediction equation. Modification of Diet in Renal Disease Study Group. Ann Intern Med. 1999;130(6):461-470. doi:10.7326/0003-4819-130-6-199903160-00002
