# Supplementary material for: Systematic review of international studies evaluating MDRD and CKD-EPI estimated glomerular filtration rate (eGFR) equations in Black adults
Source: PLoS One. 2022 Oct 18;17(10):e0276252. doi: 10.1371/journal.pone.0276252 (PMC9578594; doi:10.1371/journal.pone.0276252)
Supplement: S2 File — (DOCX) [file pone.0276252.s002.docx]

**S5 Tables and Text. Studies evaluating CKD-EPI_Cr-Cys_ equations with and without race adjustment**

Table 1A. Studies evaluating bias, accuracy, and precision of CKD-EPI_Cr-Cys_ with and without race adjustment

|  |  |  | **Bias^a^** | | **Accuracy** | | **Precision** | |
| --- | --- | --- | --- | --- | --- | --- | --- | --- |
| **Reference and eGFR equation** | **Population; #Black participants** | **mGFR** | **With race adjustment** | **Without race adjustment** | **With race adjustment** | **Without race adjustment** | **With race adjustment** | **Without race adjustment** |
| Atta, 2021^1^ | Individuals who are HIV-positive and HIV-negative; n=327 | Iohexol | HIV positive:  Bias (95% CI):  2.6 (1.0- 4.2)  HIV negative:  Bias (95% CI):  4.6 (2.4-6.8) | HIV positive:  Bias (95% CI):  -4.0 (-5.5- -2.4)  HIV negative:  Bias (95% CI):  -2.6 (-4.7- -0.5) | HIV positive:  P30, % (95% CI):  88.1 (86-90)  HIV negative:  P30, % (95% CI):  90.0 (87-93) | HIV positive:  P30, % (95% CI):  90.7 (89-93)  HIV negative:  P30, % (95% CI):  94.2 (92-97) | NR | NR |
| Bukabau, 2019^2^ | Mix: individuals with and without CKD; n=494 | Iohexol | Absolute bias (SD): 7.8 (6.2 to 9.4) | Absolute bias (SD): 1.1 (-0.4  to 2.5) | P30, % (95% CI):76.7 (73.0 to 80.5) | P30, % (95% CI):  79.2 (75.6 to 82.7) | Absolute SD:  18.1 | Absolute SD:  16.6 |
| Rocha, 2020^3^ | Patients with CKD; n=61 | ^51^Cr-EDTA | Absolute bias (IQR):  2.4 (−2.2-5.8) | Absolute bias (IQR):  −0.2 (−7.1-3.0) | P30 (95% CI): 78.7 (66.7-87.2) | P30 (95% CI):  80.3 (68.5-88.5) | Median precision (IQR):  4.3 (2.4-11.8) | Median precision (IQR):  5.5 (2.0-11.4) |
| Seape, 2016^4^ | Patients with HIV who are ART-naïve; n=97 | ^51^Cr-EDTA | Proportional bias, % (95% CI): 11.5 (5.4-17.6)  Median bias (95% CI): 8.4 (4.4-12.7) | Proportional bias, % (95% CI): 2.9 (-2.9-8.8)  Median bias (95% CI): -0.7 (-4.5-3.4) | P15, %: 44.0  P30, %: 73.0 | P15, %: 51.0  P30, %: 78.0 | Median precision, SD, 95% limit of agreement:  30.3 (-47.8- 70.9) | Median precision, SD,  95% limit of agreement:  29.2 (-54.3-60.2) |

^a^Bias differences were calculated as eGFR- mGFR (units in ml/min per 1.73 m^2^)

Abbreviations: 51Cr-EDTA=chromium-51 labeled ethylenediamine tetraacetic acid; ART=antiretroviral therapy; CI=confidence interval; CKD=chronic kidney disease; CKD-EPI=Chronic Kidney Disease Epidemiology Collaboration; Cr=creatinine; Cys = cystatin C; eGFR=estimated glomerular filtration rate; HIV=human immunodeficiency virus; IQR=interquartile range; mGFR=measured glomerular filtration rate; NR=not reported; P15=percent of eGFR values with 15% of mGFR values; P30=percent of eGFR values within 30% of mGFR values; SD = standard deviation

Description of Table 1A results:

Three of 4 studies evaluating bias of CKD-EPI_Cr-Cys_ [2-4], reported improvements with removal of the race coefficient. In the remaining study from the U.S., removal of race adjustment increased bias of the CKD-EPI_Cr-Cys_ equation among HIV-positive adults and decreased bias among HIV-negative adults; however, statistical significance was not reported [1]. In all 4 studies evaluating accuracy of CKD-EPI_Cr-Cys_ [1-4], including one U.S. study [1] of HIV-positive and -negative adults, accuracy improved with removal of race adjustment. Of 3 studies assessing precision of CKD-EPI­_Cr-Cys_ equations [2-4], the standard deviation of bias decreased with removal of race adjustment in 2 studies [2,4], and the IQR increased in 1 study of Brazilian patients with CKD [3].

Table 1B. Correlation or concordance of CKD-EPI_Cr-Cys_, studies with and without race adjustment

| **Reference** | **Population; # Black participants** | **mGFR** | **With race adjustment** | **Without race adjustment** |
| --- | --- | --- | --- | --- |
| Bukabau, 2019^2^ | Mix: individuals with and without CKD; n=494 | Iohexol | Lin’s CCC, (95%CI):  0.80 (0.75 to 0.83) | Lin’s CCC, (95%CI):  0.83 (0.79 to 0.86) |
| Rocha, 2020^3^ | Patients with CKD; n=61 | ^51^Cr-EDTA | Concordance, % (95% CI): 59.0 (46.5-70.5) | Concordance, % (95% CI): 62.3 (49.7-73.4) |

Description of Table 1B results:

In 2 studies assessing concordance or correlation between CKD-EPI_Cr-Cys_ and mGFR, improvement was observed with removal of the race coefficient [2-3]. In one study of individuals with and without CKD from the Democratic Republic of Congo and Ivory Coast, Lin’s Concordance Correlation Coefficient increased from 0.80 to 0.83 with removal of race adjustment [2]. In another study of Brazilian patients with CKD, concordance was 59.0% with race adjustment, and 62.3% without race adjustment [3].

**References**

1. Atta MG, Zook K, Brown TT, Vaidya D, Tao X, Maier P, et al. Racial adjustment adversely affects glomerular filtration estimates in Black Americans living with HIV. J Am Soc Nephrol. 2021;32(9):2143-2147. doi:10.1681/ASN.2021030311
2. Bukabau JB, Yayo E, Gnionsahé A, Monnet D, Pottel H, Cavalier E, Nkodila A, Makulo JRR, Mokoli VM, Lepira FB, Nseka NM, Krzesinski JM, Sumaili EK, Delanaye P. Performance of creatinine- or cystatin C-based equations to estimate glomerular filtration rate in sub-Saharan African populations. Kidney Int. 2019 May;95(5):1181-1189. doi: 10.1016/j.kint.2018.11.045
3. Rocha AD, Garcia S, Santos AB, Eduardo JCC, Mesquita CT, Lugon JR, et al. No race-ethnicity adjustment in CKD-EPI equations is required for estimating glomerular filtration rate in the Brazilian population. Int J Nephrol. 2020;2020:2141038. doi:10.1155/2020/2141038
4. Seape T, Gounden V, van Deventer HE, Candy GP, George JA. Cystatin C- and creatinine-based equations in the assessment of renal function in HIV-positive patients prior to commencing Highly Active Antiretroviral Therapy. Ann Clin Biochem. 2016;53(Pt 1):58-66. doi:10.1177/0004563215579695
