# Supplementary material for: Systematic review of international studies evaluating MDRD and CKD-EPI estimated glomerular filtration rate (eGFR) equations in Black adults
Source: PLoS One. 2022 Oct 18;17(10):e0276252. doi: 10.1371/journal.pone.0276252 (PMC9578594; doi:10.1371/journal.pone.0276252)
Supplement: S1 Table — (DOCX) [file pone.0276252.s003.docx]

**S1 Table. Systematic review protocol**

| **Field** | **Description** |
| --- | --- |
| **Review title** | Systematic review of international studies evaluating estimated glomerular filtration rate (eGFR) equations in Black adults |
| **Review question** | How well does eGFR, with and without race adjustment, predict measured GFR in Black adults across the full range of measured GFR in the United States and internationally? |
| **Searches** | *Databases*: PubMed, EMBASE, Web of Science, ScienceDirect, Google Scholar  *Restrictions*: English, French, Spanish, and Portuguese language articles, publication period: 1999 – present  *Date of Searches*:  PubMed: 10/23/2020  EMBASE: 10/23/2020  Web of Science: 10/23/2020  ScienceDirect: 10/23/2020  Google Scholar: 10/23/2020  Searches will be re-run prior to final analysis for any further studies identified and retrieved for potential inclusion in the systematic review.  All searches will take into account published comments and retractions. |
| **Condition or domain being studied** | Accurate assessment of kidney function is essential for proper diagnosis, staging, and management of chronic kidney disease, referral for kidney transplant, and for guiding decisions about the dosing of drugs excreted by the kidney & use of contrast for imaging studies; calculation of estimated glomerular filtration rate (eGFR) is typically used to assess kidney function.  Commonly used eGFR equations include race (Black vs. non-Black) as a coefficient, which has been estimated to increase eGFR by between 16-21% in Black patients.  The appropriateness of including the race coefficient in eGFR algorithms has increasingly become a topic of research given the increased recognition that race is a sociopolitical, rather than biological, construct. |
| **Participants / Populations** | *Inclusion*: Black adults undergoing glomerular filtration rate assessment via estimating equations.  *Exclusion*: Individuals under the age of 18. |
| **Interventions / Exposures** | Kidney function assessment via the MDRD or CKD-EPI equations with or without the inclusion of the race adjustment. |
| **Comparator(s) / Control(s)** | Comparison of multiple estimated GFR equations and/or comparison of an estimated GFR equation to a measured GFR. |
| **Types of studies to be included** | Study types to be included are validation studies and comparison studies. All other study types are excluded, though some studies not meeting inclusion criteria may be retained for review of references. |
| **Context** | Studies in all countries inclusive of clinical and research environments. |
| **Main outcome(s) and Measures of Effect** | - Type of eGFR test used (such as MDRD, CKD-EPI) - eGFR as determined with the race coefficient in Black adults - eGFR as determined without the race coefficient in Black adults - mGFR as measured in Black adults - bias, precision, and accuracy of eGFR (such as the proportion of patients with eGFR values within 30% of measured GFR) - kappa agreements and correlations between eGFR values |
| **Additional outcome(s)** | - *reporting of social determinants of health*: income, occupation, employment, educational level, diet, lifestyle choices, and/or poverty-related environmental effects - *reporting of non-GFR determinants of creatinine*: underlying co-morbidities (e.g., liver disease, anorexia, chronic illnesses), sickle cell disease, use of medications that can lead to a false elevation of creatinine, use of medications that can interfere with the assay for measuring creatinine (e.g., antibiotics, barbiturates, chemotherapeutic agents) |
| **Data extraction (selection and coding)** | *Study selection*:   - Two reviewers will independently screen abstracts and full-text for inclusion and will be blinded to each other’s decisions. Disagreements between reviewers will be resolved using an independent third reviewer. - Screening results & decisions will be collected via a structured REDCap^®^ form   *Data extraction*:   - Two independent reviewers will read the full-text of each paper selected after screening. - Disagreements on inclusion will be resolved using an independent third reviewer. - Data to be extracted from each study will include publication citation details, country of study, aim of study, total participants, population characteristics (e.g., demographics, kidney disease status, social determinants of health), study eligibility criteria, eGFR and mGFR calculation and measurement methods, main outcomes, non-GFR determinants of plasma concentrations of creatinine, study results of performance measures and levels of statistical significance. - Missing data will be sought from study investigators |
| **Risk of bias (quality) assessment** | Risk of bias will be assessed using the QUADAS-2, a tool used for diagnostic and validation studies. For studies in which there are disagreements, an adjudicator will be used who has not been directly involved in screening. |
| **Strategy for data synthesis** | A narrative synthesis will summarize conclusions per outcome and will compare study data on eGFR performance & selection by region, and population characteristics. |
| **Analysis of subgroups of subsets** | Not applicable |
| **Type and method of review** | Diagnostic systematic review |
| **Language** | English |
| **Country** | United States |
| **Organizational affiliation of the review** | Vanderbilt University Medical Center  University of Nigeria Teaching Hospital, Ituku-Ozalla  Vanderbilt University School of Medicine  Brigham and Women’s Hospital  NYU Langone Medical Center  McGaw Medical Center of Northwestern University |
| **Review team members & organizational affiliations** | ***Ms. Taneya Y. Koonce***, Center for Knowledge Management, Vanderbilt University Medical Center, United States  ***Dr. Ebele M. Umeukeje***, Division of Nephrology and Hypertension, Department of Medicine, Vanderbilt University Medical Center, United States  ***Dr. Sheila V. Kusnoor***, Center for Knowledge Management, Vanderbilt University Medical Center, United States  ***Dr. Ifeoma Isabella UIasi***, Renal Unit, Department of Medicine, College of Medicine, University of Nigeria/University of Nigeria Teaching Hospital, Ituku-Ozalla*,* Nigeria  ***Dr. Sophia Kostelanetz***, Division of General Internal Medicine and Public Health, Vanderbilt University Medical Center, United States  ***Ms. Annette M. Williams***, Center for Knowledge Management, Vanderbilt University Medical Center, United States  ***Ms. Mallory N. Blasingame***, Center for Knowledge Management, Vanderbilt University Medical Center, United States  ***Ms. Marcia I. Epelbaum***, Center for Knowledge Management, Vanderbilt University Medical Center, United States  ***Dr. Dario A. Giuse***, Department of Biomedical Informatics, Vanderbilt University Medical Center, United States  ***Dr. Annie N. Apple***, Vanderbilt University School of Medicine, United States  ***Ms. Karampreet Kaur***, Vanderbilt University School of Medicine, United States  ***Dr. Tavia González Peña***, Vanderbilt University School of Medicine, United States  ***Dr. Danika Barry***, Department of Obstetrics & Gynecology, McGaw Medical Center of Northwestern University, United States  ***Dr. Leo Eisenstein***, Department of Medicine, NYU Langone Medical Center, United States  ***Dr. Cameron T. Nutt***, Department of Medicine, Brigham and Women’s Hospital, United States  ***Dr. Nunzia Bettinsoli Giuse***, Center for Knowledge Management, Department of Biomedical Informatics, Department of Medicine, Vanderbilt University Medical Center, United States |
| **Conflicts of interest** | Not applicable |
