# Supplementary material for: Systematic review of international studies evaluating MDRD and CKD-EPI estimated glomerular filtration rate (eGFR) equations in Black adults
Source: PLoS One. 2022 Oct 18;17(10):e0276252. doi: 10.1371/journal.pone.0276252 (PMC9578594; doi:10.1371/journal.pone.0276252)
Supplement: S3 Table — (DOCX) [file pone.0276252.s005.docx]

**S4 Table. Summary characteristics of studies not included in the systematic review synthesis**

The studies by Bukabau (2018), Sagou Yayo (2016), and Yayo (2018) are not included in the systematic review analysis due to duplicate patient data with the Bukabau (2019) paper that is analyzed in the review. The paper by van Deventer et al. (2011) uses the same patient population as the van Deventer (2008) paper included in the analysis. All other publications in this list met the systematic review criteria but did not have data directly comparing eGFR to mGFR both with and without the race adjustment variable.

|  | **First Author, Year** | **Country** | **Study Design/Data Source** | **Population** | **Black participants,**  **n/N (%)** | **Method of race definition** | **eGFR equation(s)** | **mGFR** | **Outcomes reported for Black adults** |
| --- | --- | --- | --- | --- | --- | --- | --- | --- | --- |
| 1 | Akhimiona, 2018^1^ | USA | Retrospective chart review | Kidney donor candidates | 24/210  (11.4%) | Not reported | CKD-EPI_Cr_ (with 1.212 coefficient for race)  Calculated with race adjustment | ^125^I-iothalamate | Bias |
| 2 | Bukabau, 2018^2^ | Democratic Republic of the Congo | Cross-sectional; part of a larger study | Healthy individuals | 93/93 (100%) | Not reported | CKD-EPI_Cr_, CKD-EPI_Cr-Cys_, MDRD 4-variable equation (with 175 constant)  All equations calculated with and without race adjustment | Iohexol | Bias, P_10_ and P_30_  RMSE, Lin’s Concordance Correlation Coefficient, Spearman correlation |
| 3 | Coresh, 2019^3^ | USA | Cross-sectional; used data from AASK | Patients from the AASK cohort (African Americans with hypertension) | 188/188 (100%) | Self-reported | CKD-EPI_Cr ,_ CKD-EPI_Cys_, CKD-EPI_Cr-Cys_, AASK with demographics, AASK without creatinine, AASK without creatinine and demographics  AASK equations to not include a race variable; all CKD-EPI equations calculated with race correction | ^125^I-iothalmate (AASK) | Bias, precision (RMSE), 1-P_30_ |
| 4 | Currin, 2021^4^ | South Africa | Validation; used patients from ARK study | Community-dwelling with broad spectrum of kidney function | 674/674  (100%) | Not reported | CKD-EPI_Cr_, MDRD  Both equations calculated without race adjustment | Iohexol | Bias, IQR, P_30_, Spearman rank correlation, Lin’s concordance correlation |
| 5 | Flamant, 2013^5^ | France | Observational study | Patients with CKD | 302/604 (50%) | Not reported | CKD-EPI_Cr_ (with 1.08 coefficient for race)  Calculated with race adjustment | ^51^Cr-EDTA | Bias, precision, P_30_ |
| 6 | Garg, 2020^6^ | USA | Retrospective | Kidney donor candidates | 80/769  (10.4%) | Not reported | CKD-EPI_Cr_  Calculated with race adjustment | ^125^I-iothalamate | Bias, P_10_, P_30_ |
| 7 | Inker, 2018^7^ | USA | Validation; used patients from larger study | Community-based sample without CVD or prevalent CKD at baseline | 139/294 (47.3%) | Self-reported | CKD-EPI_Cr_, CKD-EPI_Cr-Cys_  Both equations calculated with race adjustment | Iohexol | Bias, IQR difference, 1-P_30_, RMSE |
| 8 | Kwong, 2010^8^ | USA | Retrospective; used data from AASK | Patients with CKD | 949/949 (100%) | Not reported | CKD-EPI_Cr-Cys_ CKD-EPI_Cys_, MDRD  Both equations calculated with race adjustment | ^125^I-iothalamate | 1-P_30_, RMSE |
| 9 | Lane, 2010^9^ | USA | Retrospective | Nephrectomy patients | 31/425  (7.3%) | Not reported | CKD-EPI_Cr_  Calculated with race adjustment | ^125^I-iothalamate | P_30_, Lin’s concordance coefficient, Pearson’s correlation coefficient^1^ |
| 10 | Lewis, 2001^10^ | USA | Cross-sectional comparison; used data from AASK | Patients with hypertensive nephrosclerosis | 1703/1703 (100%) | Self-reported | AASK 3-variable, AASK 5-variable, MDRD 4-variable equation (with 175 constant)  AASK equations do not include a race variable; MDRD calculated with race adjustment | ^125^I-iothalamate | Bias, Pearson correlation |
| 11 | Lucas, 2020^11^ | USA | Cross-sectional & longitudinal cohort; used patients from previous study | HIV-positive and HIV-negative individuals | 327/361  (91%) | Not reported | CKD-EPI_Cr_  Calculated with race adjustment | Iohexol | Bias, P_30_ |
| 12 | Sagou Yayo, 2016^12^ | Ivory Coast | Cross-sectional | Blood donors | 120/120 (100%) | Not reported | CKD-EPI_Cr_, MDRD re-expressed 4-variable equation (with 175 constant)  Both equations calculated with and without race adjustment | Iohexol | Bias, P_30_, concordance |
| 13 | Sood, 2019^13^ | USA | Comparative | Patients with hypertensive kidney disease | 652/652 (100%) | Self-reported | CKD-EPI_Cr_  Calculated with race adjustment | ^125^I-iothalamate | Bias, IQR, P_30_, correlation |
| 14 | Stevens, 2007^14^ | USA | Cross-sectional, validation; pooled data | Mix: data extracted are for AASK cohort only, which included patients with hypertension and renal dysfunction | Across all studies: 1749/5504 (32%)  AASK: 1205/1205 (100%) | Not reported | MDRD re-expressed 4- variable equation (with 175 constant)  Calculated with race adjustment | ^125^I-iothalamate | Bias, precision, P_30_ |
| 15 | Stevens, 2008^15^ | USA, France | Validation; pooled data from MDRD, AASK, CSG, and clinical population in France | Mix: included patients with hypertension, CKD, diabetes mellitus, and potential kidney donors | 1808/3418 (53%) | Varies by study | CKD-EPI_Cr_, CKD-EPI_Cr-Cys_  Both equations calculated with race adjustment | ^125^I-iothalamate or ^51^Cr-EDTA | Bias, P_30_, RMSE |
| 16 | Stevens, 2009^16^ | USA | Comparative; used pooled data | Diverse participants with a range of clinical characteristics (includes patients with and without kidney disease) | 1740/5504 (32%) | Varies by study | MDRD 4-variable equation (with 175 constant)  Calculated with race adjustment | ^125^I-iothalamate | Concordance |
| 17 | Stevens, 2010^17^ | USA | Validation; pooled data | Mix: included patients with and without kidney disease | 384/3896 (10%)^2^ | Varies by study | CKD-EPI_Cr-Cys_  Calculated with race adjustment | ^125^I-iothalamate | Bias, RMSE |
| 18 | Stevens, 2010^18^ | USA | Comparative study; used pooled data | Diverse participants with a range of clinical characteristic | 384/3896 (10%) | Varies by study | CKD-EPI_Cr_, MDRD 4-variable equation (with 175 constant)  Both equations calculated with the race adjustment | ^125^I-iothalamate | Bias |
| 19 | Stevens, 2011^19^ | USA, Europe, China, Japan, South Africa | Observational; used data from previous studies | Diverse participants with a range of clinical characteristic (includes patients with diabetes and transplant patients) | 2585/8254 (31.3%) | Varies by study | CKD-EPI_Cr,_ CKD-EPI_Cr_ with 4-level race (Black, Asian, Native American & Hispanic, Other)  Both equations calculated with race adjustment | ^125^I-iothalamate | Bias, precision (RMSE), P_30_, concordance |
| 20 | Thomson, 2020^20^ | United Kingdom | Retrospective | Kidney donors | 114/997  (11%) | Not reported | CKD-EPI_Cr ,_ MDRD 4-variable equation (with 175 constant)  Both equations calculated with race adjustment | Tc EDTA-GFR^3^ | Bias, RMSE, P_30_ |
| 21 | Van Deventer, 2011^21^ | South Africa | Prospective, comparative | Patients with established CKD or at risk of CKD | 50/50 (100%) | Not reported | CKD-EPI_Cr_, MDRD 4-variable equation (with 175 constant)  CKD-EPI calculated with and without race correction;  MDRD calculated without race adjustment | ^51^Cr-EDTA | Bias, RMSE, P_15_, P_30_, Pearson correlation, limits of agreement |
| 22 | Yayo, 2018^22^ | Ivory Coast | Prospective | Blood donors | 237/237(100%) | Not reported | CKD-EPI_Cr_  Calculated with and without race adjustment | Iohexol | Concordance |

^1^Precision assessed by Lin and Pearson correlation coefficients

^2^The numbers shown here are for the external validation set. The study also included development (n=5504; 32% Black) and internal validation (n=2750; 31%) sets.

^3^Conference abstract reports this as Tc-EDTA but this appears to be a mistake; is likely either ^99m^Tc-DTPA or ^51^Cr-EDTA.

Abbreviations: ^99m^Tc-DTPA=technetium-99m diethylenetriamine pentaacetic acid; ^51^Cr-EDTA=chromium-51 labeled ethylenediamine tetraacetic acid; AASK=African American Study of Kidney Disease and Hypertension; ARK=African Research on Kidney; CKD=chronic kidney disease; Cr=creatinine; CSG=Collaborative Study Group; CVD=cardiovascular disease; Cys=cystatin C; eGFR=estimated glomerular filtration rate; GFR=glomerular filtration rate; HIV=human immunodeficiency virus; IQR=interquartile range; MDRD=Modification of Diet in Renal Disease; mGFR=measured glomerular filtration rate; P_10_=percent of eGFR values within 10% of mGFR values; P_15_ = percent of eGFR values within 15% of mGFR values; P_30_=percent of eGFR values within 30% of mGFR values; RMSE=root mean square error

**References**

1. Akhimiona CO, Nguyen DT, Graviss EA, Gaber AO, Suki WN. Suitability of estimated glomerular filtration rate for live kidney donor selection. Transplant Proc. 2018 Dec;50(10):3071–5. doi:10.1016/j.transproceed.2018.09.013
2. Bukabau JB, Sumaili EK, Cavalier E, Pottel H, Kifakiou B, Nkodila A, et al. Performance of glomerular filtration rate estimation equations in Congolese healthy adults: the inopportunity of the ethnic correction. PloS One. 2018;13(3):e0193384. doi:10.1371/journal.pone.0193384
3. Coresh J, Inker LA, Sang Y, Chen J, Shafi T, Post WS, et al. Metabolomic profiling to improve glomerular filtration rate estimation: a proof-of-concept study. Nephrol Dial Transplant. 2019 May 1;34(5):825–33. doi: 10.1093/ndt/gfy094
4. Currin S, Gondwe M, Mayindi N, Chipungu S, Khoza B, Khambule L, et al. Evaluating chronic kidney disease in rural South Africa: comparing estimated glomerular filtration rate using point-of-care creatinine to iohexol measured GFR. Clin Chem Lab Med. 2021;59(8):1409-1420. doi:10.1515/cclm-2020-1882
5. Flamant M, Vidal-Petiot E, Metzger M, Haymann JP, Letavernier E, Delatour V, et al. Performance of GFR estimating equations in African Europeans: basis for a lower race-ethnicity factor than in African Americans. Am J Kidney Dis. 2013;62(1):182-184. doi:10.1053/j.ajkd.2013.03.015
6. Garg N, Snyder G, Li J, Mandelbrot D, Poggio ED. Performance of creatinine clearance and estimated GFR in assessing kidney function in living donor candidates. Transplantation. 2020 Mar;104(3):575–82. doi: 10.1097/TP.0000000000002797
7. Inker LA, Levey AS, Tighiouart H, Shafi T, Eckfeldt JH, Johnson C, et al. Performance of glomerular filtration rate estimating equations in a community-based sample of Blacks and Whites: the Multiethnic Study of Atherosclerosis. Nephrol Dial Transplant. 2018 Mar 1;33(3):417–25. doi: 10.1093/ndt/gfx042
8. Kwong Y-TD, Stevens LA, Selvin E, Zhang YL, Greene T, Van Lente F, et al. Imprecision of urinary iothalamate clearance as a gold-standard measure of GFR decreases the diagnostic accuracy of kidney function estimating equations. Am J Kidney Dis. 2010 Jul;56(1):39–49. doi: 10.1053/j.ajkd.2010.02.347
9. Lane BR, Demirjian S, Weight CJ, Larson BT, Poggio ED, Campbell SC. Performance of the Chronic Kidney Disease-Epidemiology study equations for estimating glomerular filtration rate before and after nephrectomy. J Urol. 2010 Mar;183(3):896–901. doi: 10.1016/j.juro.2009.11.023
10. Lewis J, Agodoa L, Cheek D, Greene T, Middleton J, O’Connor D, et al. Comparison of cross-sectional renal function measurements in African Americans with hypertensive nephrosclerosis and of primary formulas to estimate glomerular filtration rate. Am J Kidney Dis. 2001 Oct;38(4):744–53. doi: 10.1053/ajkd.2001.27691
11. Lucas GM, Atta MG, Zook K, Vaidya D, Tao X, Maier P, et al. Cross-sectional and longitudinal performance of creatinine- and cystatin C-based estimating equations relative to exogenously measured glomerular filtration rate in HIV-positive and HIV-negative persons. J Acquir Immune Defic Syndr. 2020 Dec 1;85(4):e58–66. doi: 10.1097/QAI.0000000000002471
12. Sagou Yayo É, Aye M, Konan JL, Emième A, Attoungbre ML, Gnionsahé A, et al. Inadequacy of the African-American ethnic factor to estimate glomerular filtration rate in an African general population: results from Côte d’Ivoire. Nephrol Ther. 2016;12(6):454-459. doi:10.1016/j.nephro.2016.03.006
13. Sood R, Surapaneni A, Luo S, Appel LJ, Winkler C, Grams ME, et al. Sickle cell trait, estimated glomerular filtration rate, and risk of adverse outcomes in chronic kidney disease. Am J Hematol. 2019 Oct;94(10):E275–8. doi: 10.1002/ajh.25588
14. Stevens LA, Manzi J, Levey AS, Chen J, Deysher AE, Greene T, et al. Impact of creatinine calibration on performance of GFR estimating equations in a pooled individual patient database. Am J Kidney Dis. 2007 Jul;50(1):21–35. doi: 10.1053/j.ajkd.2007.04.004
15. Stevens LA, Coresh J, Schmid CH, Feldman HI, Froissart M, Kusek J, et al. Estimating GFR using serum cystatin C alone and in combination with serum creatinine: a pooled analysis of 3,418 individuals with CKD. Am J Kidney Dis. 2008 Mar;51(3):395–406. doi: 10.1053/j.ajkd.2007.11.018
16. Stevens LA, Nolin TD, Richardson MM, Feldman HI, Lewis JB, Rodby R, et al. Comparison of drug dosing recommendations based on measured GFR and kidney function estimating equations. Am J Kidney Dis. 2009 Jul;54(1):33–42. doi: 10.1053/j.ajkd.2009.03.008
17. Stevens LA, Schmid CH, Zhang YL, Coresh J, Manzi J, Landis R, et al. Development and validation of GFR-estimating equations using diabetes, transplant and weight. Nephrol Dial Transplant. 2010 Feb;25(2):449–57. doi: 10.1093/ndt/gfp510
18. Stevens LA, Schmid CH, Greene T, Zhang YL, Beck GJ, Froissart M, et al. Comparative performance of the CKD Epidemiology Collaboration (CKD-EPI) and the Modification of Diet in Renal Disease (MDRD) Study equations for estimating GFR levels above 60 mL/min/1.73 m2. Am J Kidney Dis. 2010 Sep;56(3):486–95. doi: 10.1053/j.ajkd.2010.03.026
19. Stevens LA, Claybon MA, Schmid CH, Chen J, Horio M, Imai E, et al. Evaluation of the Chronic Kidney Disease Epidemiology Collaboration equation for estimating the glomerular filtration rate in multiple ethnicities. Kidney Int. 2011 Mar;79(5):555–62. doi: 10.1038/ki.2010.462
20. Thomson T, Kousios A, Charif R, Orr H, Dulku H, Loucaidou M. Demographic variability of kidney function in live donors: a single-centre analysis. In: Journal of the American Society of Nephrology [Internet]. Virtual United States; 2020 [cited 2021 Dec 9]. p. 741. Available from: https://www.asn-online.org/api/download/?file=/education/kidneyweek/archives/KW20Abstracts.pdf
21. van Deventer HE, Paiker JE, Katz IJ, George JA. A comparison of cystatin C- and creatinine-based prediction equations for the estimation of glomerular filtration rate in Black South Africans. Nephrol Dial Transplant. 2011;26(5):1553-1558. doi:10.1093/ndt/gfq621
22. Yayo E, Ayé M, Yao C, Gnionsahé A, Attoungbré ML, Cavalier E, et al. Measured (and estimated) glomerular filtration rate: reference values in West Africa. Nephrol Dial Transplant. 2018;33(7):1176-1180. doi:10.1093/ndt/gfx244
