# Supplementary material for: Systematic review of international studies evaluating MDRD and CKD-EPI estimated glomerular filtration rate (eGFR) equations in Black adults
Source: PLoS One. 2022 Oct 18;17(10):e0276252. doi: 10.1371/journal.pone.0276252 (PMC9578594; doi:10.1371/journal.pone.0276252)
Supplement: S4 Table — (DOCX) [file pone.0276252.s006.docx]

**S6 Table. Correlation or concordance tables for CKD-EPI_Cr_, and MDRD studies with and without race adjustment**

| **Reference** | **Population; # Black participants** | **mGFR** | **With race adjustment** | **Without race adjustment** |
| --- | --- | --- | --- | --- |
| ***CKD-EPI_Cr_ equations*** | | | | |
| Arlet, 2012^1^ | Patients with sickle cell disease;  n=64 | Iohexol | Pearson correlation coefficient: r: -0.23, p=0.06 | Pearson correlation coefficient: r: -0.43, p<0.001 |
| Bukabau, 2019^2^ | Mix: individuals with and without CKD; n=494 | Iohexol | Lin’s CCC, (95%CI):  0.71 (0.66 to 0.76) | Lin’s CCC, (95% CI):  0.81 (0.76 to 0.84) |
| Holness, 2020^3^ | Mix: patients with CKD, potential kidney donors, healthy volunteers;  n=80 | ^99m^Tc-DTPA | Agreement (95% LOA) of eGFR with mGFR: -14.5-55.2 | Agreement (95% LOA) of eGFR with mGFR: -17.9- 35.7 |
| Moodley, 2018^4^ | Inpatients and outpatients; mix of conditions; n=188 | ^99m^Tc-DTPA | Correlation, R^2^:  Female: 0.83; Male: 0.86  ROC AUC for GFR <90 ml/min/1.73 m^2^:  Female: 0.894; Male: 0.915  ROC AUC for GFR <60 ml/min/1.73 m^2^*:*  Female: 0.936; Male: 0.941 | Correlation, R^2^:  Female: 0.83; Male: 0.86  ROC AUC for GFR <90  ml/min/1.73 m^2^:  Female: 0.894; Male: 0.915  ROC AUC for GFR <60 ml/min/1.73 m^2^*:*  Female: 0.936; Male: 0.941 |
| Rocha, 2020^5^ | Patients with CKD; n=61 | ^51^CrEDTA | Concordance, % (95% CI): 52.5 (40.2-64.5) | Concordance, % (95% CI): 54.1 (41.7-66.0) |
| Wyatt, 2013^6^ | Individuals with HIV who are ART-naïve and ambulatory; n=99 | Iohexol (dried blood spots) | Variance inflation: 0.72  Correlation between eGFR and mGFR: 0.23 | Variance inflation: 0.72  Correlation between eGFR and mGFR: 0.23 |
| ***MDRD equations*** | | | | |
| Arlet, 2012^1^ | Patients with sickle cell disease; n=64 | Iohexol | Pearson’s correlation co-efficient: 0.68 | Pearson’s correlation co-efficient: 0.538 |
| Bukabau, 2019^2^ | Mix: individuals with and without CKD; n=494 | Iohexol | Lin’s CCC, (95%CI):  0.70 (0.61 to 0.77) | Lin’s CCC, (95% CI):  0.73 (0.67 to 0.78) |
| Wyatt, 2013^6^ | Individuals with HIV who are ART-naïve and ambulatory; n=99 | Iohexol (dried blood spots) | Variance inflation: 1.40  Correlation between eGFR and mGFR: 0.23 | Variance inflation: 1.40  Correlation between eGFR and mGFR: 0.23 |

Abbreviations: 51Cr-EDTA=chromium-51 labeled ethylenediamine tetraacetic acid; 99mTc-DTPA=technetium-99m diethylenetriamine pentaacetic acid; ART=antiretroviral therapy; AUC = area under the curve; CI=confidence interval; CKD=chronic kidney disease; Cr=creatinine; eGFR=estimated glomerular filtration rate; GFR=glomerular filtration rate; HIV=human immunodeficiency virus; LOA=limits of agreement; LCCC = Lin’s Concordance Correlation Coefficient; mGFR=measured glomerular filtration rate; ROC=receiver operating characteristic

**References**

1. Arlet JB, Ribeil JA, Chatellier G, Eladari D, De Seigneux S, Souberbielle JC, et al. Determination of the best method to estimate glomerular filtration rate from serum creatinine in adult patients with sickle cell disease: a prospective observational cohort study. BMC Nephrol. 2012;13:83. doi:10.1186/1471-2369-13-83
2. Bukabau JB, Yayo E, Gnionsahé A, Monnet D, Pottel H, Cavalier E, Nkodila A, Makulo JRR, Mokoli VM, Lepira FB, Nseka NM, Krzesinski JM, Sumaili EK, Delanaye P. Performance of creatinine- or cystatin C-based equations to estimate glomerular filtration rate in sub-Saharan African populations. Kidney Int. 2019 May;95(5):1181-1189. doi: 10.1016/j.kint.2018.11.045
3. Holness JL, Bezuidenhout K, Davids MR, Warwick JM. Validation of equations to estimate glomerular filtration rate in South Africans of mixed ancestry. South Afr Med J Suid-Afr Tydskr Vir Geneeskd. 2020;110(3):229-234. doi:10.7196/SAMJ.2020.v110i3.13995
4. Moodley N, Hariparshad S, Peer F, Gounden V. Evaluation of the CKD-EPI creatinine based glomerular filtration rate estimating equation in Black African and Indian adults in KwaZulu-Natal, South Africa. Clin Biochem. 2018;59:43-49. doi:10.1016/j.clinbiochem.2018.06.014
5. Rocha AD, Garcia S, Santos AB, Eduardo JCC, Mesquita CT, Lugon JR, et al. No race-ethnicity adjustment in CKD-EPI equations is required for estimating glomerular filtration rate in the Brazilian population. Int J Nephrol. 2020;2020:2141038. doi:10.1155/2020/2141038
6. Wyatt CM, Schwartz GJ, Owino Ong'or W, Abuya J, Abraham AG, Mboku C, et al. Estimating kidney function in HIV-infected adults in Kenya: comparison to a direct measure of glomerular filtration rate by iohexol clearance. PloS One. 2013;8(8):e69601. doi:10.1371/journal.pone.0069601
