# Supplementary material for: Systematic review of international studies evaluating MDRD and CKD-EPI estimated glomerular filtration rate (eGFR) equations in Black adults
Source: PLoS One. 2022 Oct 18;17(10):e0276252. doi: 10.1371/journal.pone.0276252 (PMC9578594; doi:10.1371/journal.pone.0276252)
Supplement: S5 Table — (DOCX) [file pone.0276252.s007.docx]

**S7 Table. QUADAS-2 risk of bias and applicability assessment of studies not included in the systematic review synthesis**

Responses options are as follows: signaling questions (Y, N, U); risk of bias (H, L, U); applicability (H, L, U).

| First Author, Year | Patient Selection | | | | | Index Test | | | | Reference Standard | | | | Flow and Timing | | | | |
| --- | --- | --- | --- | --- | --- | --- | --- | --- | --- | --- | --- | --- | --- | --- | --- | --- | --- | --- |
|  | Bias | | | | **Applicability concerns** | Bias | | | **Applicability concerns** | Bias | | | **Applicability concerns** | Bias | | | | |
|  | **Signaling questions** | | | **Risk of bias** |  | **Signaling questions** | | **Risk of bias** |  | **Signaling questions** | | **Risk of bias** |  | **Signaling questions** | | | | **Risk of bias** |
|  | Was a consecutive or random sample of patients enrolled? (1a.1) | Was a case-control design avoided? (1a.2) | Did the study avoid inappropriate exclusions? (1a.3) | Could the selection of patients have introduced bias? (1a.4) | Is there concern that the included patients do not match the review question? (1b.1) | Were the index test(s) results interpreted without knowledge of the results of the reference standard? (2a.1) | If a threshold was used, was it pre-specified? (2a.2) | Could the conduct or interpretation of the test(s) have introduced bias? (2a.3) | Is there concern the index test(s), its conduct, or interpretation differ from the review question? (2b.1) | Is the reference standard likely to correctly classify the target condition? (3a.1) | Were the reference standard results interpreted without knowledge of the results of the index test? (3a.2) | Could the reference standard, its conduct or interpretation have introduced bias? (3a.3) | Are there concerns that the target condition as defined by the reference standard does not match the review question? (3b.1) | Was there an appropriate interval between index test(s) and reference standard? (4a.1) | Did all patients receive a reference standard? (4a.2) | Did patients receive the same reference standard? (4a.3) | Were all patients included in the analysis? (4a.4) | Could the patient flow have introduced bias? (4a.5) |
| Akhimiona, 2018^1^ | U | Y | Y | **U** | **L** | U | Y | **U** | **L** | Y | U | **U** | **L** | U | Y | Y | Y | **U** |
| Bukabau, 2018^2^ | U | Y | Y | **U** | **L** | U | N/A | **U** | **L** | Y | U | **U** | L | Y | Y | Y | **Y** | **L** |
| Coresh, 2019^3^ | U | Y | Y | **U** | **L** | U | U | **U** | **L** | Y | U | **U** | L | U | Y | N | **Y** | **U** |
| Currin, 2021^4^ | Y | Y | Y | **L** | **L** | U | Y | **U** | **L** | Y | U | **U** | **L** | Y | Y | Y | N | **U** |
| Flamant, 2013^5^ | N | Y | U | **U** | **L** | U | N/A | **U** | **L** | Y | U | **U** | **L** | Y | Y | Y | **Y** | **L** |
| Garg, 2020^6^ | U | Y | U | **U** | **L** | U | N/A | **U** | **L** | Y | U | **U** | **L** | U | Y | Y | N | **U** |
| Inker, 2018^7^ | Y | Y | Y | **L** | **L** | U | Y | **U** | **L** | Y | U | **U** | **L** | Y | Y | N | Y | **U** |
| Kwong, 2010^8^ | N | Y | Y | **U** | **L** | U | N/A | **U** | **L** | Y | U | **U** | **L** | Y | Y | Y | Y | **L** |
| Lane, 2010^9^ | U | Y | U | **U** | **L** | U | Y | **U** | **L** | Y | U | **U** | **L** | U | Y | U | Y | **U** |
| Lewis, 2001^10^ | U | Y | Y | **U** | **L** | U | N/A | **U** | **L** | Y | U | **U** | **L** | Y | Y | Y | Y | **L** |
| Lucas, 2020^11^ | U | Y | Y | **U** | **L** | U | N/A | **U** | **L** | Y | U | **U** | **L** | Y | Y | N | Y | **U** |
| Sagou Yayo, 2016^12^ | Y | Y | Y | **U** | **L** | U | N/A | **U** | **L** | Y | U | **U** | L | U | Y | Y | **Y** | **U** |
| Sood, 2019^13^ | U | N | Y | **U** | **L** | U | N/A | **U** | **L** | Y | U | **U** | **L** | Y | Y | Y | Y | **L** |
| Stevens, 2007^14^ | N | Y | U | **U** | **L** | U | L | **U** | **L** | Y | U | **U** | **L** | U | Y | Y | Y | **L** |
| Stevens, 2008^15^ | N | Y | U | **U** | **L** | U | N/A | **U** | **L** | Y | U | **U** | **L** | U | Y | N | Y | **U** |
| Stevens, 2009^16^ | N | Y | Y | **U** | **L** | U | N/A | **U** | **L** | Y | U | **U** | **L** | U | Y | Y | Y | **U** |
| Stevens, 2010^17^ | N | Y | U | **U** | **L** | U | Y | **U** | **L** | Y | U | **U** | **L** | U | Y | N | N | **U** |
| Stevens, 2010^18^ | N | Y | Y | **U** | **L** | U | Y | **U** | **L** | Y | U | **U** | **L** | U | Y | N | Y | **U** |
| Stevens, 2011^19^ | N | Y | Y | **U** | **L** | U | Y | **U** | **L** | Y | U | **U** | **L** | U | Y | N | Y | **U** |
| Thomson, 2020^20^ | U | Y | Y | **U** | **L** | U | N/A | **U** | **L** | Y | U | **U** | **L** | U | Y | Y | Y | **U** |
| Van Deventer, 2011^21^ | U | Y | Y | **U** | **L** | U | U | **U** | **L** | Y | U | **U** | **L** | Y | Y | Y | **Y** | **L** |
| Yayo, 2018^22^ | U | Y | Y | **U** | **L** | U | N/A | **U** | **L** | Y | U | **U** | **L** | U | Y | Y | **Y** | **U** |

Abbreviations: H= High risk; L= Low risk; N= No; U=Unclear; Y = Yes, N/A = Not applicable

**Supplemental References**

1. Akhimiona CO, Nguyen DT, Graviss EA, Gaber AO, Suki WN. Suitability of estimated glomerular filtration rate for live kidney donor selection. Transplant Proc. 2018 Dec;50(10):3071–5. doi:10.1016/j.transproceed.2018.09.013
2. Bukabau JB, Sumaili EK, Cavalier E, Pottel H, Kifakiou B, Nkodila A, et al. Performance of glomerular filtration rate estimation equations in Congolese healthy adults: the inopportunity of the ethnic correction. PloS One. 2018;13(3):e0193384. doi:10.1371/journal.pone.0193384
3. Coresh J, Inker LA, Sang Y, Chen J, Shafi T, Post WS, et al. Metabolomic profiling to improve glomerular filtration rate estimation: a proof-of-concept study. Nephrol Dial Transplant. 2019 May 1;34(5):825–33. doi: 10.1093/ndt/gfy094
4. Currin S, Gondwe M, Mayindi N, Chipungu S, Khoza B, Khambule L, et al. Evaluating chronic kidney disease in rural South Africa: comparing estimated glomerular filtration rate using point-of-care creatinine to iohexol measured GFR. Clin Chem Lab Med. 2021;59(8):1409-1420. doi:10.1515/cclm-2020-1882
5. Flamant M, Vidal-Petiot E, Metzger M, Haymann JP, Letavernier E, Delatour V, et al. Performance of GFR estimating equations in African Europeans: basis for a lower race-ethnicity factor than in African Americans. Am J Kidney Dis. 2013;62(1):182-184. doi:10.1053/j.ajkd.2013.03.015
6. Garg N, Snyder G, Li J, Mandelbrot D, Poggio ED. Performance of creatinine clearance and estimated GFR in assessing kidney function in living donor candidates. Transplantation. 2020 Mar;104(3):575–82. doi: 10.1097/TP.0000000000002797
7. Inker LA, Levey AS, Tighiouart H, Shafi T, Eckfeldt JH, Johnson C, et al. Performance of glomerular filtration rate estimating equations in a community-based sample of Blacks and Whites: the Multiethnic Study of Atherosclerosis. Nephrol Dial Transplant. 2018 Mar 1;33(3):417–25. doi: 10.1093/ndt/gfx042
8. Kwong Y-TD, Stevens LA, Selvin E, Zhang YL, Greene T, Van Lente F, et al. Imprecision of urinary iothalamate clearance as a gold-standard measure of GFR decreases the diagnostic accuracy of kidney function estimating equations. Am J Kidney Dis. 2010 Jul;56(1):39–49. doi: 10.1053/j.ajkd.2010.02.347
9. Lane BR, Demirjian S, Weight CJ, Larson BT, Poggio ED, Campbell SC. Performance of the Chronic Kidney Disease-Epidemiology study equations for estimating glomerular filtration rate before and after nephrectomy. J Urol. 2010 Mar;183(3):896–901. doi: 10.1016/j.juro.2009.11.023
10. Lewis J, Agodoa L, Cheek D, Greene T, Middleton J, O’Connor D, et al. Comparison of cross-sectional renal function measurements in African Americans with hypertensive nephrosclerosis and of primary formulas to estimate glomerular filtration rate. Am J Kidney Dis. 2001 Oct;38(4):744–53. doi: 10.1053/ajkd.2001.27691
11. Lucas GM, Atta MG, Zook K, Vaidya D, Tao X, Maier P, et al. Cross-sectional and longitudinal performance of creatinine- and cystatin C-based estimating equations relative to exogenously measured glomerular filtration rate in HIV-positive and HIV-negative persons. J Acquir Immune Defic Syndr. 2020 Dec 1;85(4):e58–66. doi: 10.1097/QAI.0000000000002471
12. Sagou Yayo É, Aye M, Konan JL, Emième A, Attoungbre ML, Gnionsahé A, et al. Inadequacy of the African-American ethnic factor to estimate glomerular filtration rate in an African general population: results from Côte d’Ivoire. Nephrol Ther. 2016;12(6):454-459. doi:10.1016/j.nephro.2016.03.006
13. Sood R, Surapaneni A, Luo S, Appel LJ, Winkler C, Grams ME, et al. Sickle cell trait, estimated glomerular filtration rate, and risk of adverse outcomes in chronic kidney disease. Am J Hematol. 2019 Oct;94(10):E275–8. doi: 10.1002/ajh.25588
14. Stevens LA, Manzi J, Levey AS, Chen J, Deysher AE, Greene T, et al. Impact of creatinine calibration on performance of GFR estimating equations in a pooled individual patient database. Am J Kidney Dis. 2007 Jul;50(1):21–35. doi: 10.1053/j.ajkd.2007.04.004
15. Stevens LA, Coresh J, Schmid CH, Feldman HI, Froissart M, Kusek J, et al. Estimating GFR using serum cystatin C alone and in combination with serum creatinine: a pooled analysis of 3,418 individuals with CKD. Am J Kidney Dis. 2008 Mar;51(3):395–406. doi: 10.1053/j.ajkd.2007.11.018
16. Stevens LA, Nolin TD, Richardson MM, Feldman HI, Lewis JB, Rodby R, et al. Comparison of drug dosing recommendations based on measured GFR and kidney function estimating equations. Am J Kidney Dis. 2009 Jul;54(1):33–42. doi: 10.1053/j.ajkd.2009.03.008
17. Stevens LA, Schmid CH, Zhang YL, Coresh J, Manzi J, Landis R, et al. Development and validation of GFR-estimating equations using diabetes, transplant and weight. Nephrol Dial Transplant. 2010 Feb;25(2):449–57. doi: 10.1093/ndt/gfp510
18. Stevens LA, Schmid CH, Greene T, Zhang YL, Beck GJ, Froissart M, et al. Comparative performance of the CKD Epidemiology Collaboration (CKD-EPI) and the Modification of Diet in Renal Disease (MDRD) Study equations for estimating GFR levels above 60 mL/min/1.73 m2. Am J Kidney Dis. 2010 Sep;56(3):486–95. doi: 10.1053/j.ajkd.2010.03.026
19. Stevens LA, Claybon MA, Schmid CH, Chen J, Horio M, Imai E, et al. Evaluation of the Chronic Kidney Disease Epidemiology Collaboration equation for estimating the glomerular filtration rate in multiple ethnicities. Kidney Int. 2011 Mar;79(5):555–62. doi: 10.1038/ki.2010.462
20. Thomson T, Kousios A, Charif R, Orr H, Dulku H, Loucaidou M. Demographic variability of kidney function in live donors: a single-centre analysis. In: Journal of the American Society of Nephrology [Internet]. Virtual United States; 2020 [cited 2021 Dec 9]. p. 741. Available from: https://www.asn-online.org/api/download/?file=/education/kidneyweek/archives/KW20Abstracts.pdf
21. van Deventer HE, Paiker JE, Katz IJ, George JA. A comparison of cystatin C- and creatinine-based prediction equations for the estimation of glomerular filtration rate in Black South Africans. Nephrol Dial Transplant. 2011;26(5):1553-1558. doi:10.1093/ndt/gfq621
22. Yayo E, Ayé M, Yao C, Gnionsahé A, Attoungbré ML, Cavalier E, et al. Measured (and estimated) glomerular filtration rate: reference values in West Africa. Nephrol Dial Transplant. 2018;33(7):1176-1180. doi:10.1093/ndt/gfx244
